# Immunosuppression in tumor immune microenvironment and its optimization from CAR-T cell therapy

**DOI:** 10.7150/thno.76854

**Published:** 2022-08-29

**Authors:** Zaoqu Liu, Zhaokai Zhou, Qin Dang, Hui Xu, Jinxiang Lv, Huanyun Li, Xinwei Han

**Affiliations:** 1 Department of Interventional Radiology, The First Affiliated Hospital of Zhengzhou University, Zhengzhou, Henan 450052, China; 2 Interventional Institute of Zhengzhou University, Zhengzhou, Henan 450052, China; 3 Interventional Treatment and Clinical Research Center of Henan Province, Zhengzhou, Henan 450052, China; 4 Department of Pediatric Urology, The First Affiliated Hospital of Zhengzhou University, Zhengzhou, Henan 450052, China; 5 Department of Colorectal Surgery, The First Affiliated Hospital of Zhengzhou University, Zhengzhou, Henan 450052, China; 6 Department of Gastroenterology, The First Affiliated Hospital of Zhengzhou University, Zhengzhou, Henan 450052, China

**Keywords:** Tumor immune microenvironment, immunotherapy, chimeric antigen receptor T cell, immunosuppression network, solid tumors

## Abstract

Chimeric antigen receptor (CAR)-T cell therapy represents a landmark advance in personalized cancer treatment. CAR-T strategy generally engineers T cells from a specific patient with a new antigen-specificity, which has achieved considerable success in hematological malignancies, but scarce benefits in solid tumors. Recent studies have demonstrated that tumor immune microenvironment (TIME) cast a profound impact on the immunotherapeutic response. The immunosuppressive landscape of TIME is a critical obstacle to the effector activity of CAR-T cells. Nevertheless, every cloud has a silver lining. The immunosuppressive components also shed new inspiration on reshaping a friendly TIME by targeting them with engineered CARs. Herein, we summarize recent advances in disincentives of TIME and discuss approaches and technologies to enhance CAR-T cell efficacy via addressing current hindrances. Simultaneously, we firmly believe that by parsing the immunosuppressive components of TIME, rationally manipulating the complex interactions of immunosuppressive components, and optimizing CAR-T cell therapy for each patient, the CAR-T cell immunotherapy responsiveness for solid malignancies will be substantially enhanced, and novel therapeutic targets will be revealed.

## 1. Introduction

The past decade has witnessed a revolutionary evolution in the application of immunology to oncology treatment. Immunotherapy has made striking advances in malignancies, far outperforming conventional chemotherapy and radiotherapy. Frustratingly, the immunosuppressive properties of the tumor microenvironment (TME) pose the challenge of limited clinical efficacy and severe side effects to tumor immunotherapy 1. Priming the tumor immune microenvironment (TIME) is a critical next step in scaling up the success of current immunotherapies 2. Emerging evidence suggests that the effectiveness of immunotherapy can be maximized by addressing the tumor immunosuppressive microenvironment 3, 4. Hence, a more detailed dissection and description of immunosuppression in TIME and a deeper understanding of the tumor immunosuppressive profile are necessary for the development and optimization of novel and effective cancer immunotherapy.

**Figure 1:**
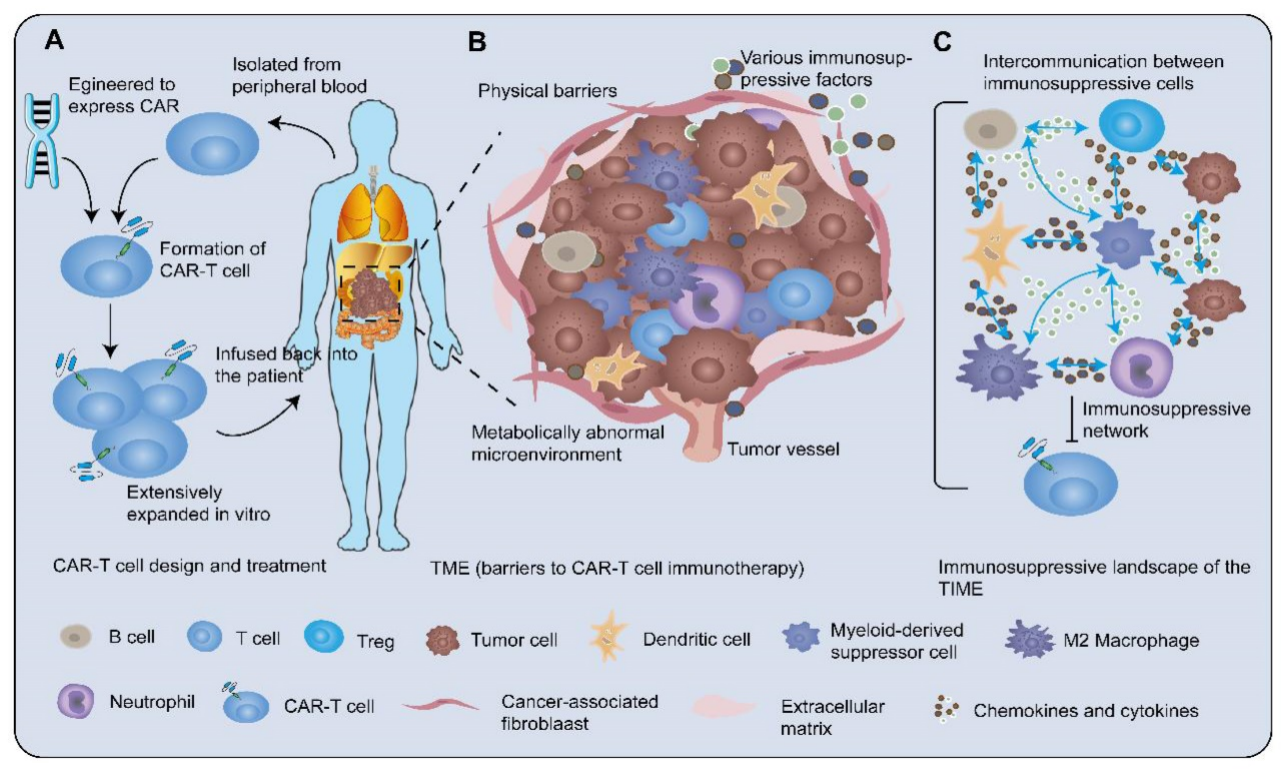
** CAR-T cell therapy and tumor immunosuppressive microenvironment. A** After isolating T cells from the peripheral blood of the patient, engineering the CARs genes into T cells to generate CAR-T cells. Then CAR-T cells are extensively expanded *in vitro* and administered to the patient. **B** TME is the central mediator of tumorigenesis and tumor-promoting function. The solid tumor microenvironment including the extracellular matrix, various immune cells, abnormal tumor vasculature, immunosuppressive molecules, and tumor metabolites prevents CAR-T cells from exerting high cytotoxicity. The tumor-associated stroma such as fibroblasts and mesenchymal cells formed physical barriers against the entry of T cells. The migration of T cells towards tumor lesions was increasingly challenged by dysregulation of adhesion molecules, mismatching of tumor-derived chemokines, and immune cell-expressed chemokine receptors. In addition, the metabolically abnormal TME impeded immune cell activity. **C** Cellular crosstalk between tumor cells and immune cells and bulk masses of immunosuppressive factors orchestrate a severely immunosuppressive tumor milieu to suppress the efficacy of CAR-T cells.

Chimeric antigen receptor (CAR)-T cell therapy is novel immunotherapy through genetically engineered T cells to express CAR targeting molecules. CAR-T cell therapy aims at introducing CARs genes into patient-derived T cells of peripheral blood in a fairly short period, where the biological properties of T cells are redirected and reprogrammed 5. T cells are rapidly expanded to derive memory and effector lymphocytes with high affinity *in vitro.* These T cells are then infused back into the patient to proliferate robustly and elicit potent anti-tumor activity **(Figure 1A)**. These synthetic receptors recognize their corresponding specific antigens using a single-chain variable fragment (scFv) from the variable region in a major histocompatibility complex-independent manner. The majority of scFv possesses binding properties similar to antibodies. CAR-T cell therapy has shown dramatic clinical responses and high rates of complete remission in hematologic malignancies 6. However, therapeutic effects in solid tumors are not durable, partly owing to physical barriers, cancer heterogeneity, and TIME, which lead to recurrent CAR antigen loss and rapid CAR-T cell exhaustion 7. Recent strategies constantly focus on harnessing CAR-T cell therapy via engineering CARs, T cells, and interactions with other elements of TIME. Among these, remodeling TIME is one of the most attractive strategies for promoting the endogenous immune response to achieve a permanent CAR-T cell engagement 8.

The immune characteristics of the tumor microenvironment (TME) have been categorized as one of the ten tumor characteristics 9, which play a decisive role in predicting the clinical outcomes of patients 10. Recognizing the essence of TIME has paramount implications for battling cancer cells. Here, we review evidence for each of the following perspectives. Firstly, how tumors orchestrate an immunosuppressive microenvironment promoting immune tolerance and evasion. Secondly, how to enhance the effectiveness of CAR-T cell therapy. Ultimately, we further elucidate the utilization of refreshing technologies to reverse the immunosuppressive microenvironment.

## 2. The Intricate Network Sustaining Immunosuppression in TIME

TME contributes to unfavorable immunotherapy efficacy by preventing CAR-T cells from exerting high cytotoxicity against tumor cells **(Figure 1B)**. As proof: 1) The tumor-associated stroma such as fibroblasts, mesenchymal cells, and various extracellular matrices formed stumbling block against the entry of T cells 11; 2) The migration of T cells towards tumor lesions was increasingly overshadowed by the 'bad guys', such as dysregulation of adhesion molecules, aberrant tumor-related vasculature, and mismatching of chemokines and their receptors 11; 3) Cancer cells expressed ligands of suppressive immune checkpoints such as immune-dampening PD-1 ligand (PD-L1)/L2 12 and recruited more immunosuppressive cells 13, 14 to interfere with the effector T cells (Teffs) cytotoxic function. Additionally, the metabolically abnormal TME characterized by restricted nutrient availability, acidosis, and local hypoxia, impeded immune cell activity 15, 16. Nevertheless, these straitened circumstances have in turn spurred the design of CAR-T cells to better unleash the potential of immunotherapy. Generally, TME is the central mediator of tumorigenesis and tumor-promoting function, with immunological features possessing indispensable status. The complex immunosuppressive network in TME, that is tumor immunosuppression microenvironment, consists of miscellaneous immunosuppressive cell subsets, secretions, and signals that inhibit the recruitment, proliferation, differentiation, and execution of effector functions of immune cells. Here, we focus on the immunosuppressive landscape of TIME **(Figure 1C)**.

### 2.1. Representative immunosuppressive cells in TIME

**Figure 2:**
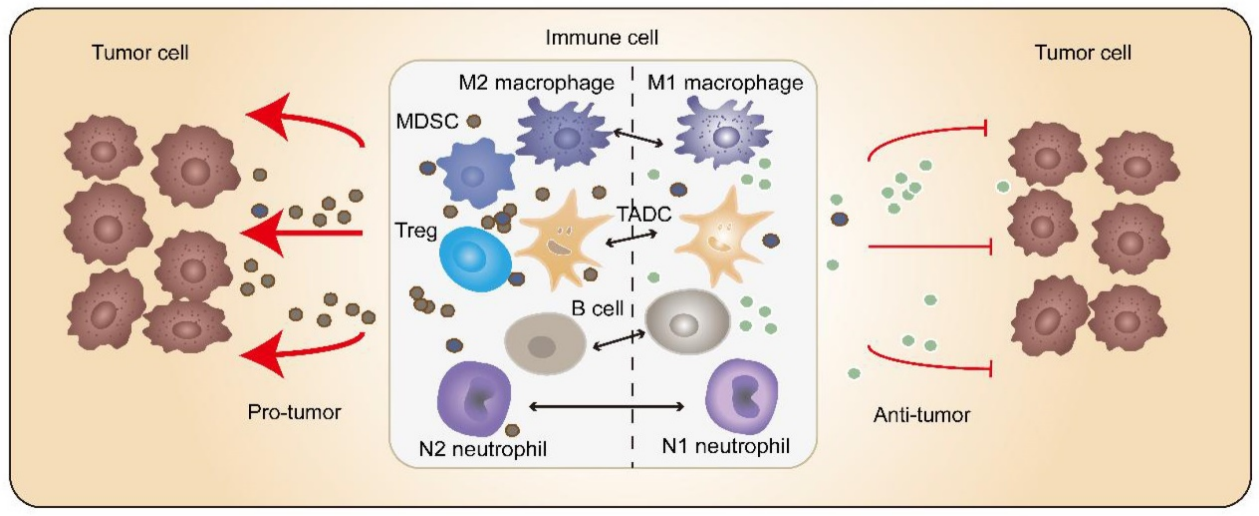
** Miscellaneous immune cells inside tumors.** The tumor-associated immune cells may possess tumor-antagonizing or tumor-promoting capacities. The homogeneous immune cells are able to change their state due to different tumor ecosystem or different stages of tumorigenesis. For instance, macrophages can shift from a hostile to a friendly status after immunotherapy. Elevating the amount of tumor-antagonizing immune cells opens a broad window for tumor treatment.

Tumor-associated immune cells possess crucial functions in tumorigenesis, which antagonize and/or promote tumors. Homogeneous immune cells could reshape themselves depending on different tumor ecosystems **(Figure 2)**.

#### 2.1.1 Myeloid-derived suppressor cells (MDSCs)

MDSCs are pathologically activated monocytes and neutrophils with vigorous immunosuppressive behavior and are implicated in the negative modulation of immune responses and poor clinical outcomes 17. An ocean of extracellular factors could induce MDSCs differentiation or expansion, encompassing granulocyte-macrophage colony-stimulating factor (GM-CSF), macrophage colony-stimulating factor (M-CSF), interferon-gamma (IFN-γ), interleukin (IL)-6, prostaglandin E2 (PGE2), IL-13, and vascular endothelial growth factor (VEGF). In contrast, IL-4 and all-trans-retinoic acid can inhibit this procedure 18, 19. MDSCs could be classified into granulocytic MDSCs (G-MDSCs) and monocytic MDSCs (M-MDSCs) according to the phenotypic and morphological characteristics or cell surface markers of MDSCs. M-MDSCs hindered CD8+ T cells via an inducible nitric oxide (iNOS)-mediated pathway 20-22, whereas G-MDSCs suppressed T-cell function through arginase and/or reactive oxygen species (ROS)-dependent mechanisms 23, 24.

Apart from promoting tumor development by forming pre-metastatic niches and angiogenesis 25, 26, MDSCs could also contribute to immunosuppression through the following mechanisms: 1) Inducting other immunosuppressive cells: MDSCs could not only secret IL-10 and transforming growth factor-β (TGF-β) to directly hamper Teffs but also induce the *de novo* generation of regulatory T cells (Tregs) mediated by IL-10 and IFN-γ *in vivo* 21. CCR5 ligands CCL5, CCL4, and CCL3, produced by tumor-infiltrating M-MDSCs could recruit massive amounts of CCR5+ Tregs 27. They could also shift macrophages from M1 to an M2-like state with immunosuppressive features. It was demonstrated that MDSCs-produced IL-10 decreased macrophage IL-6 and TNF-α 28. 2) Blocking lymphocyte homing: splenic or blood-borne MDSCs were shown to execute far-reaching immune suppression through downregulating L-selectin lymph node homing receptors on naïve T and B cells. Furthermore, loss of L-selectin expression could disrupt T cell trafficking. T cells preconditioned by MDSCs have diminished responses to subsequent antigen exposure 29, 30. 3) Engendering reactive oxygen and nitrogen species: secreting ROS such as hydrogen peroxide, hydroxyl radicals, and superoxide anions was a well-known strategy to eradicate tumor-infiltrating lymphocytes. Enhanced ROS levels could increase the quantity and quality of tumor-infiltrating MDSCs by NF erythroid 2-related factor 2 and VEGF receptors, which might create a positive feedback loop 31, 32. MDSCs could also generate high levels of reactive nitrogen species (RNS) via activating the iNOS pathway 33. RNS could induce chemokine CCL2 nitration, hinder T-cell infiltration and engage T-cell apoptosis, leading to the trapping of specific T-cells in the tumor-associated stroma 34, 35. 4) Competing with immune cells for nutrient metabolites: high expression of arginase I and cationic amino acid transporter 2B by mature MDSCs could rapidly incorporate L-Arginine (L-Arg) and deplete extracellular L-Arg *in vitro* 36. L-Arg depletion blocked antigen-specific proliferation of OT-1 and OT-2 cells and the re-expression of CD3zeta in stimulated T cells 37. 5) Another mechanism by which MDSCs inhibit immune cells included the expression of ectoenzymes regulating adenosine metabolism from ATP 38 and negative immune checkpoint molecules 39.

#### 2.1.2 Tumor-associated macrophages (TAMs)

Proverbially, macrophages are essential components of innate immunity and leukocyte infiltration present in solid tumors, which regulate cancer-related inflammation and constitute vital regulators of tumor initiation and progression. Circulating monocytes could be recruited into the tumor stroma by multiple chemokines and cytokines such as CCL2, GM-CSF, and VEGF family members 40, 41. TME then prompted the differentiation of monocytes into TAMs 42. The immunological effects of TAMs are pro- and anti-tumor functions based on the state of macrophage activation. Collectively, M1 macrophages could respond to danger signals that produced type I pro-inflammatory cytokines such as IL-1, IL-12, and TNF-α. Conversely, M2 macrophages expressed scavenger receptors and type II cytokines, such as IL-4, IL-10, and IL-13, promoting anti-inflammatory responses 43-45. M2 macrophages possessed pro-tumorigenic functions including promotion of tumor cell growth 46, drug resistance 47 and metastasis 48, neo-angiogenesis 9, 49, and immune suppression 50. The M1/M2 balance varied with cancer types. Both M1 and M2 macrophages have high levels of plasticity, with the ability to be converted into each other upon TIME or therapeutic interventions 51, 52. For example, hepatocellular carcinoma (HCC)-derived exosomes could reshape macrophages and result in M2-polarized TAMs via inducing pro-inflammatory factors and activating NF-κB signaling 53.

TAMs could regulate the immunological activity of T cells to foster tumor progression. Pro-inflammatory cytokines produced by TAMs could trigger the accumulation and expansion of CD4+ Th17 cells to foster angiogenesis and overexpressing CTLA-4, programmed death-1 (PD-1), and glucocorticoid-induced tumor necrosis factor receptor (GITR), thereby promoting tumor development 54. TAMs could also attract Tregs into tumor tissues by producing various chemokines, such as CCL17, CCL18, and CCL22 55, 56. Additionally, they could directly handicap the proliferation of CD8+ T cells through the metabolism of L-arginine and the production of iNOS and ROS 34, 57.

TGF-β in TME upregulated Tim-3 expression on TAMs, which promoted tumorigenesis and tolerance via NF-κB signaling and downstream IL-6 production 58. It has been also validated that TAMs could produce IL-6 and signal via STAT3 to facilitate the expansion of carcinoma stem cells sustaining carcinogenesis 59. Likewise, TAMs were demonstrated as a nexus with the prognosis of numerous tumors, such as HCC 60 and breast cancer 61.

#### 2.1.3 Tregs and regulatory B cells (Bregs)

Commonly referred to as Tregs are CD4+CD25+Foxp3+ T cells, which could chemoattract TME through chemokine gradients such as CCR8-CCL1, CCR4-CCL17/22. Tregs negatively moderate immune responses to maintain autoimmune tolerance and homeostasis. However, excessive suppression of immune responses in the TME promoted tumor progression 62. Abundant infiltration of Tregs positively correlated with depressed survival in various tumor types, such as pancreatic and melanoma 63. Intratumoral Tregs were highly immunosuppressive and consumed IL-2 through the high expression of CD25 (IL-2 receptor subunit-α), thereby limiting the activation and proliferation of Teffs dependent on IL-2. Tregs also inhibited and/or killed Teffs by releasing suppressive molecules and producing cytotoxic substances 64, 65. Upregulating immune checkpoint molecules including the lymphocyte activation gene-3 (LAG-3), CTLA-4, PD-1, and inducible co-stimulator (ICOS), was an alternative strategy for Tregs to suppress Teffs 64.

B cells in TME play a double-edged sword effect either by promoting tumor immunity or enhancing tumorigenesis 66. It has been shown that any B cell has the potential to differentiate into Bregs. Bregs prohibited the expansion of T cells and other immune-system pro-inflammatory lymphocytes to exert suppression. Despite partial consensus on the immunosuppressive effector functions of Bregs, the field has not yet reached a unified view on their phenotype 67. Immunohistochemical analyzes showed that the frequency of CD19+IL-10+ Bregs in tongue squamous cell carcinoma was significantly higher than adjacent normal tissue. Increased Bregs could convert CD4+CD25- T cells into CD4+Foxp3+ Tregs in cytological experiments and are associated with cancer progression and worse survival 68. Correspondingly, B cells enriched in the ascites from ovarian cancer patients were inversely correlated with the frequencies of IFN-g+CD8+ T cells, but positively correlated with Tregs 69. Another study showed that bone marrow-derived Bregs could abrogate NK cell antibody-dependent cell-mediated cytotoxicity against multiple myeloma cells 70.

#### 2.1.4 Others

Tumor-associated neutrophils (TANs) are another type of immune cell that infiltrates various tumors. TANs could be recruited to TME by IL-8 via CXCR1/CXCR2 receptors and abolish the ability of CD8+ T cells through TNF-α production-mediated NO 71, 72. Similar to TAMs, TANs are partitioned into anti-tumoral (N1) and pro-tumoral (N2) phenotypes. Type I IFNs could alter neutrophils into N1 phenotypes, which transform the pro-tumor properties of low neutrophil extracellular traps and TNF-α expression into anti-tumor milieus. The constantly changing TIME drove the polarization of TANs. Altered TANs were connected with different prognoses in cancers and modulation of TANs phenotypes may represent a potent therapeutic option 73, 74. More significantly, TANs infiltration or neutrophil-lymphocyte ratio strongly correlates with cancer development, which may have utility as a predictive biomarker to monitor cancer patients receiving immunotherapy (e.g., melanoma, metastatic renal cell cancer) 75, 76.

Mast cells (MCs) are well known to participate in allergy and inflammation. The ability of MCs in TME has been hypothesized to be either pro- or anti-tumor 77. The number of infiltrating MCs correlated positively with poor cancer prognosis 78, 79. Recently, Leveque *et al.* have demonstrated that tumor-associated mast cells (TAMCs), a heterogeneous population, harbor a distinct phenotype compared to MCs present in the non-lesional homologue of lung cancer 80. It is further noted that the TAMCs subset expressing alpha E integrin, namely CD103+ TAMCs, appeared more actively to interact with CD4+ T cells and located closer to tumor cells than their CD103- counterparts. Recruitment of TAMCs was mediated by chemotactic agents released by TME, such as VEGF, CXC chemokine ligand (CXCL)12, PGE2, and platelet-derived growth factor (PDGF) 80, 81. Activated TAMCs could express CCL5 and IL-33 to further recruit MCs into tumor sites and activate themselves in an autocrine manner. TAMCs could drive angiogenesis, immunosuppression, as well as tumor invasion and metastasis through secreting substantial proteolytic enzymes and growth factors 79, 81. Nevertheless, some studies have shown that a high frequency of TAMCs correlated with better progression-free survival and overall survival 79, 80. Together, the immunosuppression of TAMCs in TME remains relatively vague and controversial.

Dendritic cells (DCs) acting as specialized antigen-presentation cells have long been recognized as a critical factor in T-cell-mediated anti-tumor reactions. DCs uptake and cross-present tumor antigens to naïve T cells accompanied by priming and activating CTLs with the ability to eradicate tumor cells 82. Intriguingly, although tumor-associated dendritic cells (TADCs) could prevent prolongedly steady tumor expansion at early stages, the enduring activity of T cells was abrogated by microenvironmental immunosuppressive TADCs at late stages, becoming less responsive 83, 84. The tumor-antagonizing role of DCs within TME faced inauspicious roadblocks^85^. The chemotactic gradient of TME caused a paucity of DCs recruitment and a constellation of immunosuppressive factors accelerated tolerance and DCs dysfunction 86, 87. Numerous oncogenic signaling axes could restrict the ability of DCs. Ruiz *et al.* reported that HCC impaired DCs recruitment due to the absence of a tumor-derived chemokine CCL5 via tumor-intrinsic Wnt/β-catenin signaling 88. STAT3-mediated signaling also handicapped the differentiation and maturation of DCs by producing IL-10, TGF-β, and indoleamine 2,3-dioxygenase (IDO) 89. Furthermore, TADCs could express immune-inhibitory checkpoints, such as PD-L1 and TIM-3, to impede the activation of CTLs 88, 90. TADCs could also spark tumor plasticity, growth, and metastasis by promoting genomic instability, neovascularization, and immunometabolism 91, 92.

### 2.2 The intricate immune inhibitory factors network in TIME

Immune cells, endothelial, stromal, and tumor cells within the TME secreted bulk masses of immunosuppressive factors including chemokines, cytokines, and inhibitory molecules to assist and/or restrain each other, which orchestrates a severely immunosuppressive tumor milieu. These factors bind to corresponding receptors via paracrine, autocrine or endocrine means to modulate tumor growth/invasion/metastasis, and immune responses, thereby reshaping TME and mediating intercellular crosstalk. Some of their specific features have been mentioned above. Herein, we dissect the representative participants in the current data (**Table 1**).

### 2.3 Tumor antigen heterogeneity

In addition to the exogenous factors mentioned above (e.g., immunosuppressive cells and factors of the TIME), the endogenous factors (e.g., tumor antigen heterogeneity) could further exacerbate the immunosuppressive landscape of TIME. Identifying tumor-specific antigens as targets is a challenge for CAR-T cell therapy to win the war against solid tumors. The optimal target surface antigen should possess excellent coverage, high expression on solid tumors, and not affect normal cells to avoid on-target and off-tumor cross-reactions 110. However, antigenic heterogeneity is a specific feature of solid tumors. Tumor cells could conceal themselves by removing or constantly modifying their representative antigens, which are antigen loss and antigen-low escape, confusing the targeted attacks of CAR-T cells 111, 112. It has been demonstrated that antigen loss occurred via two distinct mechanisms: antigen escape or lineage switch 113. Antigen escape, or isoform switch, occurs when cancer cells present different patterns of targeted antigens without being detected by CAR-T cells. This mechanism is probably connected to gene mutations at the antigen locus due to immune pressure by CAR-T cells 114. Lineage switch is linked to substantial changes in chromatin accessibility and rewiring of transcriptional programs in tumor cells, including alternative splicing 115. Antigen-low escape indicated that CAR-T cells targeting specific antigens were unable to effectively eliminate specific antigens-low cells 116. There is an overlap between antigen escape and low antigen density escape.

## 3. Up-to-date Strategies for CAR-T Cell Therapy to Address the Hostile Immune Microenvironment

With the increasing understanding of TIME, more strategies concentrate on 're-editing' TIME to better arouse tumor-antagonizing immunity, which offers a novel tactic for CAR-T cell therapy.

### 3.1 Targeting immune suppressive cells

**Figure 3:**
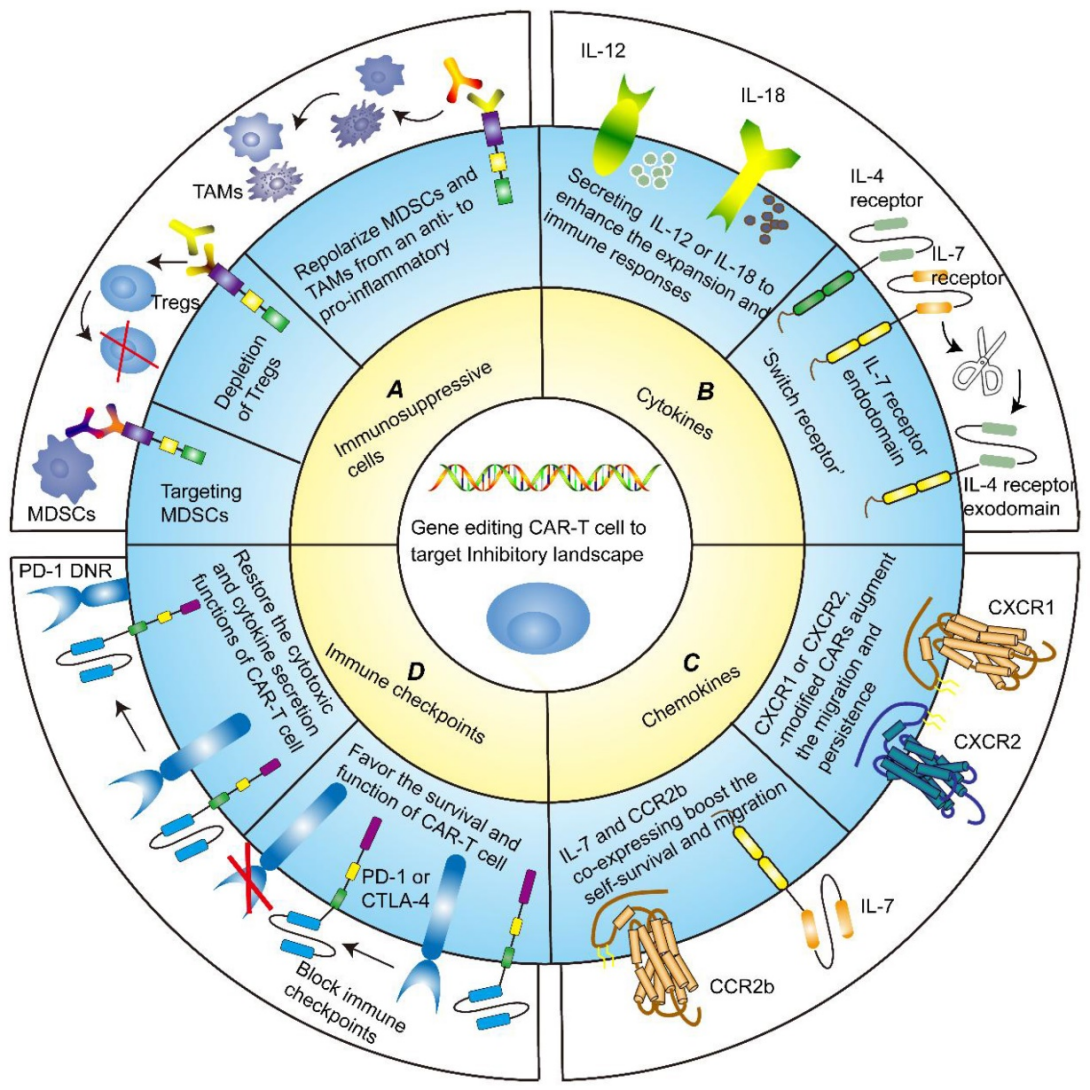
** Gene modification strategies for CAR-T cells. A** Targeting immune suppressive cells. CAR-T cells exhibit enhanced persistence and proliferation by targeting MDSCs or depleting Tregs. Likewise, repolarizing MDSCs and TAMs from an immunosuppressive to pro-inflammatory phenotype could boost the anti-tumor functions of CAR-T cells. **B** Targeting cytokines milieu. CAR-redirected T-cell engineered to release inducible IL-12 or IL-18 could eliminate the antigen-loss cancer cells, recruit immune cells and reinforce their functions, and sustain pro-inflammatory responses. An alternative strategy to counteract immunosuppressive TME is to generate an inverted cytokine receptor in which the IL-4 receptor exodomain was fused to the IL-7 receptor endodomain, which removes the side effects of the immunosuppressive cytokine IL-4. **C** Targeting chemokines milieu. CXCR1 or CXCR2-modified CARs remarkably favored T-cell migration and persistence in TME, leading to the induction of complete tumor regression and durable immunologic memory. IL-7 and CCR2b co-expressing CAR-T cells also boosted the self-survival and migration, increased IFN-γ, Gzms-B, and IL-2 expression, and inhibited tumor growth. **D** Targeting immune checkpoints. Blocking immune checkpoints sponsored CAR-T cell survival and function of killing tumor cells. Dominant-negative receptors (DNRs) could interfere with the PD-1/PD-L1 pathway, thereby restoring the cytotoxic and cytokine secretion functions of CAR-T cells.

Several approaches of targeting immunosuppressive cells to advance the tumor-killing ability of CAR-T cells have been applied in basic studies **(Figure 3A)**. It has been reported that MDSCs inhibited the cytotoxicity of different generations of disialoganglioside (GD2)-CAR-T cell. The frequency of circulating MDSCs was inversely correlated with the levels of GD2-CAR-T cell in phase I/II clinical trial, further underlining the importance of targeting immunosuppressive cells 117. Frustratingly, due to the high similarity of MDSCs to normal myeloid cells, it is extremely challenging to specifically target and eliminate MDSCs from TME without detrimental off-target signaling. Nevertheless, flow cytometry analysis confirmed that TRAIL receptor 2 (TR2) was highly expressed on the surface of MDSCs. By co-expressing a costimulatory TR2.41BB receptor to target both tumor cells and MDSCs, CAR-T cells exhibited enhanced persistence and proliferation 118.

The combination of CAR-T therapy with immunomodulatory agents was proposed for obtaining satisfactory therapeutic results 119, 120. Sun and colleagues performed co-administration of Olaparib, a poly (ADP-ribose) polymerase inhibitor killing cancer cells by participating in DNA defect repair pathways, in combination with CAR-T cells 121. The co-administration enhanced anti-tumor responses by impeding MDSCs migration through the SDF1a/CXCR4 axis in immunocompetent mouse models of breast cancer. The combination of CAR-T cell therapy with the folate-targeted Toll-like receptor 7 agonists repolarized MDSCs and TAMs from a hostile to the amicable state. Moreover, it also concurrently enhanced the accumulation and activation of both CAR-T cells and endogenous T cells 122.

Engineering CARs to target TAMs is also a pervasive experimental direction. TAMs that expressed appreciable levels of folate receptor beta (FRβ) possessed an immunosuppressive M2-like phenotype, which indicated that targeting FRβ could limit the outgrowth of solid tumors 123, 124. Subsequently, Rodriguez-Garcia and colleagues demonstrated that CAR-T cell-mediated selective elimination of FRβ+ TAMs could augment pro-inflammatory monocyte enrichment and endogenous tumor-specific CD8+ T cell influx, delayed cancer progression, and prolonged survival in syngeneic cancer mouse models 124. By employing a similar strategy, other researchers designed third-generation CAR-natural killer (NK) and -T cells. They specifically targeted human CSF 1 receptor (CSF1R) on TAMs and had favorable cytotoxicity to remove the inhibitory effect of M2 TAMs 125. However, Wu and colleagues reported that the effects of GPC3-CAR T cells could be further improved, which can at least partially be ascribed to IL12 secretion of TAMs 119. This implied that TAMs are highly plastic and heterogeneous. And to some extent, early TIME might exhibit anti-tumor effects, while late TIME was more biased toward tumor promotion 83, 84.

Likewise, depletion of other immunosuppressive cells is an attractive way. Using an anti-CD25 antibody with augmented binding to activating Fc gamma receptor (FcγR, an inhibitory receptor that blocks intra-tumoral Tregs depletion), CD25+ Tregs were depleted. This reinforced cancer-infiltrating CD8+ T cells and improved the eradication of established tumors 126. The third-(CD28-4-1BBz) generation CAR-T cell that the PYAP Lck binding-motif of CD28 domain incorporated two amino acid substitutions to eliminate IL-2-associated Tregs has been invented. CAR-T cell remarkably retarded cancer growth without a need for lymphodepletion 127. Alternatively, pretreatment of immune cells to lessen the inhibitory effect may serve as bridging salvage chemotherapy for further CAR-T cell therapy 128. For instance, daratumumab had successfully treated a case-patient with relapsed/refractory multiple myeloma-transformed plasma-cell leukemia. The immune cell subset analysis of patients revealed dramatical down-regulation of CD38+ NK cells, Tregs, and Bregs 129. In mice tumor models, CAR-T cells armed with neutrophil-activating protein (NAP, a pluripotent pro-inflammatory protein) could slow tumor occurrence and development, and bolster survival rates of innate immune cells, regardless of host haplotype, target antigen, and tumor type 130. Taken together, reprogramming cancer-associated immunosuppressive cells to a more favorable niche could provide new dawn for CAR-T cell therapy.

### 3.2 Targeting the cytokine and/or chemokine milieu

#### 3.2.1 Cytokines

Induced local dissemination of stimulatory factors could counteract the immunosuppressive dilemma to reinforce CAR-T cell potency **(Figure 3B)**. Currently, recombinant cytokine drugs such as IL-2 and IFN-α, have been approved for anti-tumor treatment. This facilitates the generation of “armored” CAR-T cells engineered to secret pro-inflammatory cytokines. To illustrate, CAR-redirected T-cells tailored to release inducible IL-12 could eliminate the antigen-loss cancer cells, recruit macrophages and reinforce the function, and sustain pro-inflammatory responses 131. Combining CAR-T cells with recombinant human IL-12 also fueled anti-cancer activity 132. IL-18-expressing CAR-T cells have also been shown to possess excellent multiplication power and induce deeper B cell aplasia in mice with B16F10 melanoma 133. Interestingly, Kunert and colleagues verified that IL-12-secreting T-cell receptor-modified T (TCR-T) cells caused severe edema-like toxicity, augmented blood levels of IFNγ and TNFα, and decreased numbers of peripheral TCR-T cells, while IL-18-secreting TCR-T cells reduced cancer burden and prolonged survival without side effects 134.

More recently, a preclinical study emphasized that CAR-T cells via autocrine IL-23 signaling had the superior anti-tumor capacity and attenuated side effects, with decreased PD-1 expression and increased granzyme B in comparison to those expressing IL-18 135. Gene-engineering expressing IL-15 136, IL-7 and CCL19 137, and IL-4/21 138 has also been explored to boost anti-tumor activities through promoting proliferation, survival, activation, and stem cell memory subset of CAR-T cells and endogenous T cells. The above CAR-T cells equipped with cytokines could reverse immunosuppressive TME not only by extending their expansion and lifespan, but also by activating immune effector cells.

Another approach to addressing the efficacy conundrum is to modify CAR-T cells by rewiring inhibitory inputs to stimulatory outputs or being refractory to inhibitory cytokines present in TME. Engineering of TGF-β CAR-T cells demonstrated that CARs could express a dominant-negative TGF-βRII (dnTGF-βRII), thereby blocking TGF-β signaling in T cells. DnTGF-βRII increased CAR-T cell proliferation and long-term *in vivo* persistence, bolstered cytokine secretion, fought against exhaustion, and induced tumor eradication 139, 140. In contrast, CAR-T cells engineered to respond to TGF-β allowed themselves to convert immunosuppressive cytokine into triggers of anti-tumor activity 139, 141. TGF-β-responsive CAR-T cells could proliferate and produce T helper type 1 (Th1)-associated cytokines in the presence of soluble TGF-β, protect nearby cells from the immunosuppressive effects of TGF-β, and significantly improve the anti-tumor efficacy of neighboring CTLs 139, 141. Notably, TGF-β was associated with the lack of immune response exhibiting low levels of T cell penetration into the tumor center 142. Thus, targeting this axis could be the most susceptible solution to destroy solid tumors.

'Switch receptor' could also achieve by rewiring negative signals. Mohammed and colleagues 143 generated an inverted cytokine receptor in which the IL-4 receptor exodomain was fused to the IL-7 receptor endodomain to remove the side effects of the immunosuppressive cytokine IL-4. This result manifested that engineered CAR-T cells could transmit inhibitory signals into therapeutic stimulants through an IL-7-induced downstream pathway and possessed strengthened proliferation and superior anti-tumor ability 143, 144. Pleasingly, combined single-cell transcriptome analysis and CRISPR-Cas9 knockin nominated a novel TGF-βR2:4-1BB switch receptor to improve CAR-T cell fitness and solid tumor clearance 145.

#### 3.2.2 Chemokines

Given the pivotal role of chemokines and their receptors in recruiting immune cells and trafficking CAR-T cells into tumors, numerous studies have endeavored to integrate chemokines and CAR-T cells to battle tumors in recent years **(Figure 3C)** 146. Most investigations focused on CXCR2-modified CAR-T cells. Preclinical models demonstrated that IL-8 receptor, CXCR1 or CXCR2, -modified CARs remarkably favored T cell migration and persistence in TME, thereby inducing complete cancer regression and immunologic memory in aggressive tumors such as ovarian, glioblastoma, and pancreatic cancer 147. CXCR2-modified CAR-T cells could also accelerate trafficking *in vivo* and tumor-specific accumulation 148. Whilding and colleagues came to a similar conclusion that CXCR2-expressing CAR-T cells efficiently migrated towards tumor-conditioned media containing IL-8 and had a more favorable toxicity profile of eliciting anti-cancer responses against αvβ6-expressing ovarian or pancreatic tumor xenografts 149.

Targeting other chemokines is under active experimentation, some of which have achieved satisfactory therapeutic effects in solid tumors. Some researchers constructed a lentivirus-based CAR gene transfer system targeting CCR4, a chemokine receptor over-expressed in T-cell malignancies and Tregs profiles 150. CCR4-expressing directed CAR-T cells displayed antigen-dependent potent cytotoxicity against T-cell malignancies in mouse xenograft models. Wang and colleagues designed co-expressing mesothelin (Msln) and chemokine receptors CCR2b or CCR4 CAR-T cells 151. The Msln-CCR2b-CAR and/or Msln-CCR4-CAR T cells possessed fortified migration and infiltration, specifically exerted cytotoxicity, and expressed high levels of pro-inflammatory cytokines. Compared with traditional CAR-T cells, IL-7 and CCR2b co-expressing CAR-T cells boosted self-survival and migration, enhanced IFN-γ, Gzms-B, and IL-2 expression, and obstructed tumor growth 152. These strategies all pave the way for the clinical application of CAR-T cells.

Undoubtedly, CAR-T cells engineered to acquire responsiveness to cytokines, chemokines, and their receptors exhibit stronger tumor-antagonizing and trafficking functions than the classical second-generation. CAR-T cells against other soluble inhibitors such as PGE2 153 and VEGF receptor-2 (VEGFR-2) 154 could also conduce to solid tumor regression, shedding light on the potential novel solid tumors treatment regimens. Even so, individuals treated with the aforementioned CAR-T cells might develop off-target effects and various systemic toxicities such as cytokine release syndrome 155, and neurotoxicity 156, even leading to death 157. It has been hypothesized that gaining insight into cytokine and chemokine expression profiles across different tumor types and different individuals and exploring how to traffic CAR-T cells into TME with personalized medicine could lessen the severity of toxicities. No matter what, the efficacy in patients is required to be further tested in clinical trials.

### 3.3 Targeting immune checkpoints

Immune checkpoint receptors and ligands, such as PD-1, PD-L1, and CTLA-4, could preclude CAR-T cell cytotoxicity and induce anergy in TME 158. Checkpoint blockades are a successful therapeutic intervention to date for solid tumors, which have been used concurrently with CAR-T cell therapy in ongoing clinical trials and obtained potent synergistic activities **(Figure 3D)** 159. Combination therapy targeting PD-1 has been demonstrated to promote the survival of CAR-T cell and kill PD-L1+ cancer cells via activation-induced cell death 160. A multitude of studies had also shown that PD-1/PD-L1 pathway interference, including anti-PD-1 antibodies, cell-intrinsic PD-1 shRNA blockades, or PD-1 dominant-negative receptors (DNRs) restored the cytotoxic and cytokine secretion functions of CAR-T cell through tailored to secrete immune-checkpoint blockades or combination therapy 159, 161, 162. Moreover, the combination therapy of GD2-CAR-T cells with checkpoint blockades was well tolerable and effective in patients with relapsed or refractory neuroblastoma 163. Likewise, clinically significant antitumor responses following PD-1 blockade combined with CAR-T cells have been reported in a patient with refractory diffuse large B-cell lymphoma and progressive lymphoma 164.

Gene-editing technologies that knock out immune checkpoints could also be applied to abrogate the expression of T cell negative regulators. PD-1 knockout through TALEN technology augmented the persistence and tumor clearance capability of intratumoral T cells and established durable anti-tumor memory 165. In addition, CRISPR-Cas9-mediated PD-1 disruption enhanced CAR-T cell cytokine production and cytotoxicity towards PD-L1+ cancer cells without attenuating the proliferation 166. Using the CRISPR/Cas9 system to disrupt universal CAR-T cells with genes lacking TCR and PD-1, Ren and colleagues demonstrated potent anti-tumor effector function *in vitro* and animal models 167. Knockout of CD3-signaling regulator diacylglycerol kinase for resistance to PGE2 could also augment CAR-T cell abilities 168. Nevertheless, the safety and feasibility of genome-editing technology to reverse checkpoint-induced inhibitory signaling in clinical patients are still in non-stop exploration.

Similar to PD-1, LAG-3 and T cell immunoglobulin and mucin-domain containing-3 (Tim-3) known as T cell exhaustion markers functioned as coinhibitory receptors to throttle T cell proliferation and cytokine production 98, 169. Shapiro *et al.* found that soluble LAG3 promoted tumor cell activation and anti-apoptotic effects 170. Afterward, blocking LAG-3 ramped up T cell activation, which rendered LAG-3 evolve into a popular target. Equivalently, targeting Tim-3 is also a prospective strategy 169. Indeed, checkpoint blockades have revolutionized the field of immuno-oncology, which could be operative against malignancies that fail CAR-T cell therapy and refuel CAR 164. However, one hypothesis suggests that CAR-T cell therapy for most tumor cells with unexpressed targeted antigen is unlikely to be successful unless combination strategies that enhance bystander effects are applied, and neither anti-PD-1/CTLA-4 antibodies stir bystander effects 171. Combination therapy could also increase on-target off-cancer toxicity. Inspiringly, Rafiq *et al.* modified CAR-T cells to secrete PD-1-blocking scFv 172. ScFv-secreting CAR-T cells were equally effective or superior to combination therapy with CAR-T cells and a checkpoint inhibitor. Notably, this strategy could prevent the toxicities connected with systemic checkpoint inhibition.

### 3.4 Targeting tumor antigen heterogeneity

In the previous section on targeting immunosuppressive cells and/or factors, we covered part of targeting tumor antigen heterogeneity. Admittedly, as CAR-T cells targeting solid tumors become increasingly effective due to tumor antigen heterogeneity, clinical outcomes will be constrained 112. Single-target CAR-T cells typically lead to positive selection of antigen-mismatched tumor cells, thereby dampening durable efficacy. Conversely, bispecific CAR-T cells can launch a dual attack on evading cancer cells to avoid ineffective treatment due to heterogeneous target antigen expression and overgrowth of cancers lost a single targeted antigen 173. CAR monomers of bispecific CAR-T cells comprised two patterns. One was two distinct scFvs 'hand-in-hand' on one cell, whereas the other was two independent CAR monomers with distinct scFvs on a single cell. Recent studies showed that bispecific CAR-T cells could enhance tumor-suppression capacity through dual antigen recognition and internal activation to effectively eradicate tumor cells 174, 175.

Another way to achieve multiple targets is to create bispecific T cell engagers (BiTEs) linking CD3 scFvs and tumor-associated antigens. BiTEs are bispecific antibodies that redirect T cells to target antigen-expressing tumors. Compared with single-target CAR-T cells, BiTE CAR-T cells demonstrated prominent activation, cytokine production, and cytotoxicity in response to target-positive tumors 176, 177. Choi *et al.* developed a bicistronic construct to drive CAR expression specific for EGFRvIII and EGFR. Unlike EGFR-specific CAR-T cells, BiTE-CAR efficiently redirected CAR-T cells, recruited untransduced bystander T cells against heterogeneous tumors, and was not toxic to human skin grafts *in vivo* 177. In conclusion, BiTEs secreted by T cells exhibit robust anti-tumor function, substantial sensitivity, and specificity, establishing a bridge between CAR-T cells and solid tumors 178.

## 4. Future Directions for CAR-T Cell to Optimize TIME

Despite countless researchers working on the clinical translation of CAR-T cells to overcome the unfriendly TIME, there are numerous hard nuts to crack without any clue. Table 2 lists the ongoing clinical trials of CAR-T cell therapy in solid tumors and no results for all studies in clinical applications. Solid tumors excel at selectively fascinating or avoiding leukocyte subsets and inducing dysfunctional or immunosuppressive phenotypes on resident leukocytes to promote immunosuppression and/or tumor progression 8. The road for CAR-T cells to conquer the chilly TIME of solid tumors is extremely tortuous. Several reports indicated that aggressive cancer growth was primarily driven by mobilization of immunosuppressive leukocytes in TME rather than loss of tumor immunogenicity following a relatively long incubation period 83, 84. Due to the spatial and temporal variability of TIME, both immune composition and tumor-specific targets undergo dynamic variation in genotype, phenotype and transcriptome, further illustrating the undisputed primacy of targeting TIME 179.

**Figure 4:**
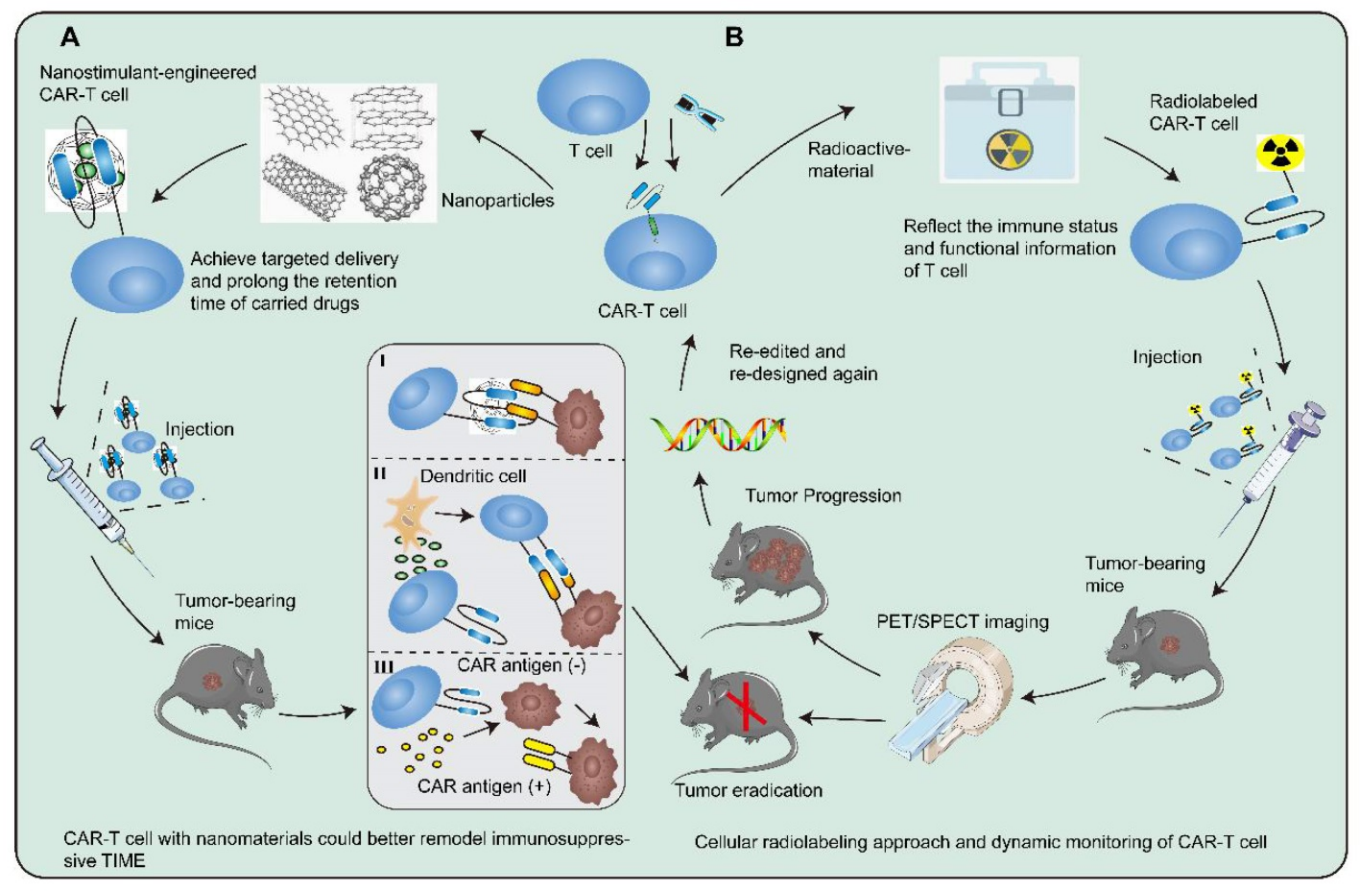
** Cutting-edge technologies to augment the security and durability of CAR-T cells.** There are increasingly revolutionary technologies and researches applied in CAR-T cell therapy, which provide a bright guide to reshaping TIME. **A** Nanoparticles could achieve targeted delivery and prolong the retention time of carried drugs. Nanostimulant-engineered CAR-T cells not only could evoke robust anti-tumor efficacy and biosafety via immunofeedback but allow for controllable drug effects on CAR-T cells. **I** CAR-T cells penetrate the tumor location and elicit the first killing. **II** Secreting pro-inflammatory factors to trigger immune cells and endogenous T cells at the right moment could initiate secondary killing and form a positive anti-tumor cycle from CAR-T cells to T cells.** III** The cancer cells with CAR antigen loss could be recognized and killed by CAR-T cells delivering peptide antigen to cancer cells. **B** Radioactive material is extensively utilized and may have distinct immunomodulatory effects both locally and systemically. The combination of radiotherapy and CAR-T cell therapy could maximize the effect of immunotherapy. The separated T cells are directly labeled and genetically modified. The radiolabeled CAR-T cells are infused into tumor-bearing mice and monitored by PET/SPECT imaging. If the tumor worsens in tumor-bearing mice, then redesign and relabel the CAR-T cells with radioactive material.

Recently, the rapid advancement of nanomedicine has provided inimitable insights into the security and durability of CAR-T cell therapy. Nanoparticles that achieve targeted delivery and prolong the retention time of carried drugs could better remodel the tumor immunosuppressive environment than traditional drugs 180. Luo and colleagues developed IL-12 nanostimulant-engineered CAR-T cell (INS-CAR-T) biohybrids that not only evoked robust anti-tumor efficacy and biosafety via immune feedback but allowed for controllable drug effects 181. INS-CAR T biohybrids enjoyed various characteristics, including elevated expression of anti-tumor factors, efficient recruitment, deep penetration, and selective proliferation. Several researchers put forward an alternative strategy that CAR-T cells engineered to produce extracellular vesicles containing RN7SL1 by delivering the pattern recognition receptor agonists could effectively stimulate anti-tumor immunity 182. These CAR-T cells with delivery systems or functional nanochaperones could be a powerful tool with the possibility to shift the immunologic landscape and outlook for solid tumors **(Figure 4A)**.

Radioactive material is extensively utilized and may have distinct immunomodulatory effects locally and systemically. In addition to boosting local expression of multiple cytokines and promoting vascular normalization, radiotherapy could activate endogenous target antigen-specific immunity to yield complementary benefits, thereby maximizing the effect of CAR-T cells 183-185. Radionuclide-based molecular imaging afforded the visualization and therapeutic monitoring of CAR-T cells through cellular radiolabeling approach or gene imaging strategies in determining whether CAR-T cells homed and infiltrated into the tumor bed, as well as their survival and persistence in TIME 186. The endogenous cell imaging could reflect the immune status and functional information of T cells and even delineate the developmental trajectory of immune cells. Dynamic monitoring will help researchers understand cellular behavior *in vivo*, allowing better infusion timing and dosage optimization to avoid potentially lethal systemic toxicity **(Figure 4B)** 187. As for assessing the prognosis and suitability of CAR-T cell therapy, types of TIME could be considered as a novel biomarker to stratify the overall survival risk of untreated tumor patients and tertiary lymphoid structure (TLS) could be used as a sign of effective immunotherapies 179, 188. Upregulation of TLS in most tumors could foster CAR-T cell immunotherapy 188.

Until recently, tumor-on-chip offered a renewed direction for preclinical CAR-T cell research. Compared with conventional *in vivo* animal models and *in vitro* planar cell models, emerging tumor-on-chip platforms integrating microfluidics, tissue engineering, and 3D cell culture have successfully mimicked the key structural and functional properties of TME *in vivo* 189-191. Several studies have attempted to generate functional immune cells from human pluripotent stem cells in combination with neo-platforms, such as the generation of T-cell progenitors from hematopoietic organoids 192 and the invention of hematopoietic organs-on-chips 193. The studies pushed the limit of pluripotent stem cells to produce immune cells useful for CAR-T therapy. Thus, tumor/organ-on-chip platforms hold great promise as more accurate and realistic models for investigating the immunosuppressive mechanisms of TIME as well as the drug toxicity and efficacy of CAR-T cell therapy before being applied to individuals. Additionally, using a comprehensive single-cell gene expression and TCR sequencing dataset for pre- and post-infusion CAR-T cells, Wilson *et al.* 194 found a unique signature of CAR-T cell effector precursors present in pre-infusion cell products. These effector precursor CAR-T cells exhibited functional superiority and decreased expression of the exhaustion-associated transcription factor, consistent with post-infusion cellular patterns observed in patients. Engineering alternative immune cells (such as NK and macrophages) to express CAR targeting molecules is also an optimal strategy. CAR-NK cells or macrophages were shown to induce a pro-inflammatory TIME and boost anti-tumor T cell activity 195, 196. Collectively, these nascent technologies or preclinical studies offer great potential for therapeutic applications.

## 5. Conclusion

CAR-T immunotherapy is revolutionizing the paradigm of cancer therapy. However, unique obstacles posed by solid tumors remain a Gordian knot for CAR-T immunotherapy. The intricate interactions among immune cells, tumor cells, immune molecules, and cytokines form TIME that throttles immune responses and encourages tumor development. By precisely mapping the complex regulatory network of TIME and drawing the optimization strategies, we could provide patients with more effective and tailored CAR-T immunotherapy. To address the hostile TIME and mitigate or even obviate the risk of serious adverse reactions, cutting-edge technologies, such as nanoparticle delivering systems, radionuclide-based molecular imaging, and novel tumor-on-chip platforms are gradually applied for CAR-T immunotherapy. Indeed, the current understanding of TIME is only the tip of an iceberg. Nevertheless, we believe that by progressively regulating the harsh TIME to reshape a friendly microenvironment, CAR-T immunotherapy will produce a more astonishing breakthrough.

## Figures and Tables

**Table 1:** Representative immune inhibitory factors mediated pro-/anti-tumor function

Molecules and signaling pathways	Category	Description	References
IL-7, IL-15, IL-21	Pro-inflammatory cytokine	**·** The common γ chain cytokine family **·** Promote the generation of the stem cell-like memory T cell phenotype **·** Boost the tumor-killing activity of NK and CTL cells	93, 94
IL-18	Pro-inflammatory cytokine	**·** A potent inducer of IFN-γ **·** Contribute to T and NK cell activation and Th-1 cell polarization **·** Participate in promoting tumor angiogenesis, metastasis, and immune escape	95, 96
IL-12	Pro-inflammatory cytokine (anti-tumor immunity modulator)	**·** Activate NK cells and T lymphocytes and induce Th-1 type responses **·** Increase IFN-γ secretion and cytotoxicity	97
IL-4, IL-13	Anti-inflammatory/inhibitory cytokine	**·** Suppress type 1 immunity and cytotoxic T cell development **·** Induce expansion of monocytes or macrophages within the tumor stroma and antagonize the tumor-suppressing activity of type 1‑activated macrophages	19, 43
IL-6/STAT3	Pro-inflammatory/carcinogenesis signaling	**·** Induce MDSCs differentiation or expansion, then result in immunosuppression **·** Favor the expansion of carcinoma stem cells sustaining carcinogenesis **·** Up-regulate IDO production; down-regulate IFN-γ; induce T cells apoptosis and dysfunction	18, 19, 59
PD-1/PD-L1	Immune checkpoint molecules	**·** Expressed on activated T lymphocytes and deliver immunosuppressive signals, drive T cells into a state of exhaustion, tolerance, or dysfunction	64
LAG3	Immune checkpoint molecule	**·** An exhaustion marker and inhibitory receptor **·** Impair CD4+ and CD8+ TILs functions	64, 98
CTLA-4	Immune checkpoint molecule	**·** Exert an inhibitory signal to T cells, and then make T cells with an inactive state **·** Enhance Tregs activity and IDO and IL-10 productions in DCs	64, 99
Tim3/Galectin-9 signaling pathway	Immune checkpoint signaling	**·** Inhibit the activation and function of CTLs and promote immune cells apoptosis	90, 98
CXCL9, CXCL10, CXCL11/CXCR3 axis	Th1-type chemokines	**·** Produced by tumors and immune cells, augment Teff and NK cell trafficking into tumors **·** Promote CTLs and NK cells differentiation, and regulate differentiation of naive T cells to T helper 1 (Th1) cells **·** Possess an opposite role in either pro- or anti-tumor responses	100, 101
CXCL12/CXCR4	C-X-C subfamily chemokine and its receptor	**·** CXCL12 contributes to the migration of plasmacytoid DCs into TME, tumor proliferation, and metastasis **·** CXCR4 can only combine with CXCL12 and prevents tumor metastasis and development	102
CCL2, CCL3, CCL5	C-C subfamily chemokines	**·** Recruit macrophages, neutrophils, and Tregs into TME, promote the polarization of M2-macrophages **·**Assist the accumulation of immunosuppressive MDSCs and tumor metastasis	27, 40, 41
CXCR1	C-X-C chemokine receptor family	**·** Increase neutrophil recruitment **·** Bolster proliferation of tumor-initiating cells, and neoplastic mass formation	72
CXCL1/CXCR2 signaling	Immunosuppressive signaling axis	**·** Stimulate tumor proliferation and self-renewal, correlate with pro-angiogenic and cancer-promoting genes of the tumor, involve in tumor metastasis **·** Amplify its production and remarkably induce both tumor-promoting and immunosuppressive factors	103
IDO	Immunosuppressive modulator	**·** Suppress T cell cytotoxicity and IL-2, IFN-γ, and TNF-α production **·** Enhance Treg-mediated immunosuppression, thus creating a tolerogenic milieu in tumor sites	50, 104
VEGF, PDGF	Growth factors	**·** Promote angiogenesis and immune evasion **·** Mediate recruitment of immune inhibitory cells and other pro-inflammatory signals in TME	32, 81
Hypoxia /HIF1α	Tumor metabolic environmental factors/ “oxygen sensor”	**·** Increase the MDSCs infiltration and suppressive function **·** Control the PD-L1 expression on cancer cells and PD-1 expression on T cells, accordingly dampening T cell survival and effector functions	16, 105
Hydrogen peroxide, hydroxyl radicals, superoxide anions, and nitric oxide	Reactive species (ROS, RNS)	**·** Lead to immune cell apoptosis and deficient effector differentiation, **·** Inhibit the proliferation and function of CD8+ T cells **·** Contribute to MDSC recruitment into the TME, result in differentiation and expansion of MDSCs	16, 32-34
Extracellular adenosine/ Adenosine signaling	immunosuppressive molecule/ signaling	**·** Impair the activation, proliferation, survival and cytokine production of T cells and other immune cells **·** Activation of adenosine receptors A2A and A2B on tumor-infiltrating immune cells suppress the anti-tumor activities of these cells **·** Favor tumor progression and escape from anti-tumor immunity	106-109

**Table 2:** Representative CAR-T cell clinical trials for solid tumors.

NCT Number	Phase	Status	Tumor types	Interventions	Study result
NCT03851146	Ⅰ	Completed	Advanced Cancer	LeY CAR-T cells	-
NCT03706326	Ⅰ | Ⅱ	Unknown status	Advanced Esophageal Cancer	Anti-MUC1 CAR-T cells	-
NCT03874897	Ⅰ	Recruiting	Advanced Solid Tumor	CAR-CLDN18.2 T-Cells	-
NCT02862028	Ⅰ | Ⅱ	Unknown status	Advanced Solid Tumor	HerinCAR-PD1 cells	-
NCT05287165	Ⅰ	Recruiting	Digestive System Neoplasms | Pancreatic Cancer | Colorectal Cancer	IM96 CAR-T cells	-
NCT05275062	Ⅰ	Recruiting	Gastric Cancer | Esophagogastric Cancer | Pancreatic Cancer	IM92 CAR-T cells	-
NCT03356795	Ⅰ | Ⅱ	Unknown status	Cervical Cancer	Cervical cancer-specific CAR-T cells	-
NCT05089266	Ⅰ	Not yet recruiting	Colorectal Cancer	αPD1-MSLN-CAR-T cells	-
NCT05415475	Ⅰ	Recruiting	Colorectal Cancer | Esophageal Cancer | Stomach Cancer | Pancreatic Cancer	CEA CAR-T cells	-
NCT04503980	Ⅰ	Recruiting	Colorectal Cancer | Ovarian Cancer	αPD1-MSLN-CAR T cells	-
NCT05341492	Ⅰ	Recruiting	Lung Cancer | Breast Cancer	EGFR/B7H3 CAR-T cells	-
NCT04581473	Ⅰ | Ⅱ	Recruiting	Gastric Adenocarcinoma | Pancreatic Cancer | Gastroesophageal Adenocarcinoma	CT041 autologous CAR-T cells	-
NCT05131763	Ⅰ	Recruiting	Hepatocellular Carcinoma | Glioblastoma | Medulloblastoma | Colon Cancer	NKG2D-based CAR-T cells	-
NCT02932956	Ⅰ	Active, not recruiting	Liver Cancer	Glypican 3-specific CAR-T cells	-
NCT04489862	Ⅰ	Recruiting	Non-small-cell Lung Cancer | Mesothelioma	αPD1-MSLN-CAR-T cells	-
NCT04864821	Ⅰ	Not yet recruiting	Osteosarcoma | Neuroblastoma | Gastric Cancer | Lung Cancer	Targeting CD276 CAR-T cells	-
NCT04981691	Ⅰ	Recruiting	Refractory Malignant Solid Neoplasm	anti-MESO CAR-T cells	-
NCT03356782	Ⅰ | Ⅱ	Recruiting	Sarcoma | Osteoid Sarcoma | Ewing Sarcoma	Sarcoma-specific CAR-T cells	-
NCT02107963	Ⅰ	Completed	Sarcoma | Osteosarcoma | Neuroblastoma | Melanoma	Anti-GD2-CAR engineered T cells	-
NCT03545815	Ⅰ	Recruiting	Solid Tumor	anti-mesothelin CAR-T cells	-
NCT05437315	Ⅰ | Ⅱ	Recruiting	Solid Tumor	bi-4SCAR GD2/PSMA T cells	-
NCT05382377	Ⅰ	Recruiting	Solid Tumor	NKG2D CAR-T cells	-
NCT05373147	Ⅰ	Recruiting	Solid Tumor	αPD1-MSLN-CAR-T cells	-
NCT04976218	Ⅰ	Recruiting	Solid Tumor | EGFR Overexpression	TGFβR-KO CAR-EGFR T Cells	-
NCT04467853	Ⅰ	Recruiting	Solid Tumors	LCAR-C18S cells	-

-: No Results Available

## Abbreviations

BiTE: bispecific T cell engager
Breg: regulatory B cell
CAR: chimeric antigen receptor
CTL: cytotoxic lymphocyte
CTLA-4: cytotoxic T lymphocyte-associated antigen 4
CXCL: CXC chemokine ligand
DC: dendritic cell
DNR: dominant-negative receptor
FcγR: Fc gamma receptor
FRβ: folate receptor beta
GD2: disialoganglioside
GITR: glucocorticoid-induced tumor necrosis factor receptor
GM-CSF: granulocyte-macrophage colony-stimulating factor
G-MDSC: granulocytic MDSC
HCC: hepatocellular carcinoma
ICOS: inducible co-stimulator
IFN-γ: interferon-gamma
IL: interleukin
iNOS: inducible nitric oxide
LAG-3: lymphocyte activation gene-3
L-Arg: L-Arginine
MC: Mast cell
M-CSF: macrophage colony-stimulating factor
MDSC: myeloid-derived suppressor cell
M-MDSC: monocytic MDSC
Msln: mesothelin
NAP: neutrophil-activating protein
NK cell: natural killer cell
Nrf2: NF erythroid 2-related factor 2
PD-1: programmed death-1
PDGF: platelet-derived growth factor
PD-L1: PD-1 ligand
PGE2: prostaglandin E2
RNS: reactive nitrogen species
ROS: reactive oxygen species
TADC: tumor-associated dendritic cell
TAM: tumor-associated macrophage
TAMC: tumor-associated mast cell
TAN: tumor associated neutrophil
TCR: T-cell receptor
Teff: effector T cell
TGF-β: transforming growth factor-β
Th1: T helper type 1
Tim-3: T cell immunoglobulin and mucin-domain containing-3
TIME: tumor immune microenvironment
TLS: tertiary lymphoid structure
TME: tumor microenvironment
TR2: TRAIL receptor 2
Treg: regulatory T cell
VEGF: vascular endothelial growth factor

## References

[1] Gupta RG, Li F, Roszik J, Lizee G (2021). Exploiting Tumor Neoantigens to Target Cancer Evolution: Current Challenges and Promising Therapeutic Approaches. Cancer Discov.

[2] van den Ende T, van den Boorn HG, Hoonhout NM, van Etten-Jamaludin FS, Meijer SL, Derks S (2020). Priming the tumor immune microenvironment with chemo(radio)therapy: A systematic review across tumor types. Biochim Biophys Acta Rev Cancer.

[3] Huang X, Han L, Wang R, Zhu W, Zhang N, Qu W (2022). Dual-responsive nanosystem based on TGF-beta blockade and immunogenic chemotherapy for effective chemoimmunotherapy. Drug Deliv.

[4] Zhao Y, Pan Y, Zou K, Lan Z, Cheng G, Mai Q (2023). Biomimetic manganese-based theranostic nanoplatform for cancer multimodal imaging and twofold immunotherapy. Bioact Mater.

[5] Walsh Z, Yang Y, Kohler ME (2019). Immunobiology of chimeric antigen receptor T cells and novel designs. Immunol Rev.

[6] Feins S, Kong W, Williams EF, Milone MC, Fraietta JA (2019). An introduction to chimeric antigen receptor (CAR) T-cell immunotherapy for human cancer. Am J Hematol.

[7] Li L, Zhu X, Qian Y, Yuan X, Ding Y, Hu D (2020). Chimeric Antigen Receptor T-Cell Therapy in Glioblastoma: Current and Future. Front Immunol.

[8] Hong M, Clubb JD, Chen YY (2020). Engineering CAR-T Cells for Next-Generation Cancer Therapy. Cancer Cell.

[9] Hanahan D, Weinberg RA (2011). Hallmarks of cancer: the next generation. Cell.

[10] Miyashita M, Sasano H, Tamaki K, Hirakawa H, Takahashi Y, Nakagawa S (2015). Prognostic significance of tumor-infiltrating CD8+ and FOXP3+ lymphocytes in residual tumors and alterations in these parameters after neoadjuvant chemotherapy in triple-negative breast cancer: a retrospective multicenter study. Breast Cancer Res.

[11] Sterner RC, Sterner RM (2021). CAR-T cell therapy: current limitations and potential strategies. Blood Cancer J.

[12] Pardoll DM (2012). The blockade of immune checkpoints in cancer immunotherapy. Nat Rev Cancer.

[13] Tanaka A, Sakaguchi S (2017). Regulatory T cells in cancer immunotherapy. Cell Res.

[14] Loeuillard E, Yang J, Buckarma E, Wang J, Liu Y, Conboy C (2020). Targeting tumor-associated macrophages and granulocytic myeloid-derived suppressor cells augments PD-1 blockade in cholangiocarcinoma. J Clin Invest.

[15] Martinez M, Moon EK (2019). CAR T Cells for Solid Tumors: New Strategies for Finding, Infiltrating, and Surviving in the Tumor Microenvironment. Front Immunol.

[16] Shen L, Xiao Y, Tian J, Lu Z (2022). Remodeling metabolic fitness: Strategies for improving the efficacy of chimeric antigen receptor T cell therapy. Cancer Lett.

[17] Veglia F, Sanseviero E, Gabrilovich DI (2021). Myeloid-derived suppressor cells in the era of increasing myeloid cell diversity. Nat Rev Immunol.

[18] Tcyganov EN, Hanabuchi S, Hashimoto A, Campbell D, Kar G, Slidel TW (2021). Distinct mechanisms govern populations of myeloid-derived suppressor cells in chronic viral infection and cancer. J Clin Invest.

[19] Zhao Y, Wu T, Shao S, Shi B, Zhao Y (2016). Phenotype, development, and biological function of myeloid-derived suppressor cells. Oncoimmunology.

[20] Youn JI, Nagaraj S, Collazo M, Gabrilovich DI (2008). Subsets of myeloid-derived suppressor cells in tumor-bearing mice. J Immunol.

[21] Huang B, Pan PY, Li Q, Sato AI, Levy DE, Bromberg J (2006). Gr-1+CD115+ immature myeloid suppressor cells mediate the development of tumor-induced T regulatory cells and T-cell anergy in tumor-bearing host. Cancer Res.

[22] Movahedi K, Guilliams M, Van den Bossche J, Van den Bergh R, Gysemans C, Beschin A (2008). Identification of discrete tumor-induced myeloid-derived suppressor cell subpopulations with distinct T cell-suppressive activity. Blood.

[23] Rodriguez PC, Ernstoff MS, Hernandez C, Atkins M, Zabaleta J, Sierra R (2009). Arginase I-producing myeloid-derived suppressor cells in renal cell carcinoma are a subpopulation of activated granulocytes. Cancer Res.

[24] Ko JS, Zea AH, Rini BI, Ireland JL, Elson P, Cohen P (2009). Sunitinib mediates reversal of myeloid-derived suppressor cell accumulation in renal cell carcinoma patients. Clin Cancer Res.

[25] Shi H, Zhang J, Han X, Li H, Xie M, Sun Y (2017). Recruited monocytic myeloid-derived suppressor cells promote the arrest of tumor cells in the premetastatic niche through an IL-1beta-mediated increase in E-selectin expression. Int J Cancer.

[26] Binsfeld M, Muller J, Lamour V, De Veirman K, De Raeve H, Bellahcene A (2016). Granulocytic myeloid-derived suppressor cells promote angiogenesis in the context of multiple myeloma. Oncotarget.

[27] Schlecker E, Stojanovic A, Eisen C, Quack C, Falk CS, Umansky V (2012). Tumor-infiltrating monocytic myeloid-derived suppressor cells mediate CCR5-dependent recruitment of regulatory T cells favoring tumor growth. J Immunol.

[28] Beury DW, Parker KH, Nyandjo M, Sinha P, Carter KA, Ostrand-Rosenberg S (2014). Cross-talk among myeloid-derived suppressor cells, macrophages, and tumor cells impacts the inflammatory milieu of solid tumors. J Leukoc Biol.

[29] Ku AW, Muhitch JB, Powers CA, Diehl M, Kim M, Fisher DT (2016). Tumor-induced MDSC act via remote control to inhibit L-selectin-dependent adaptive immunity in lymph nodes. Elife.

[30] Ostrand-Rosenberg S, Sinha P (2009). Myeloid-derived suppressor cells: linking inflammation and cancer. J Immunol.

[31] Beury DW, Carter KA, Nelson C, Sinha P, Hanson E, Nyandjo M (2016). Myeloid-Derived Suppressor Cell Survival and Function Are Regulated by the Transcription Factor Nrf2. J Immunol.

[32] Kusmartsev S, Eruslanov E, Kubler H, Tseng T, Sakai Y, Su Z (2008). Oxidative stress regulates expression of VEGFR1 in myeloid cells: link to tumor-induced immune suppression in renal cell carcinoma. J Immunol.

[33] Raber PL, Thevenot P, Sierra R, Wyczechowska D, Halle D, Ramirez ME (2014). Subpopulations of myeloid-derived suppressor cells impair T cell responses through independent nitric oxide-related pathways. Int J Cancer.

[34] Molon B, Ugel S, Del PF, Soldani C, Zilio S, Avella D (2011). Chemokine nitration prevents intratumoral infiltration of antigen-specific T cells. J Exp Med.

[35] Nagaraj S, Gupta K, Pisarev V, Kinarsky L, Sherman S, Kang L (2007). Altered recognition of antigen is a mechanism of CD8+ T cell tolerance in cancer. Nat Med.

[36] Rodriguez PC, Quiceno DG, Zabaleta J, Ortiz B, Zea AH, Piazuelo MB (2004). Arginase I production in the tumor microenvironment by mature myeloid cells inhibits T-cell receptor expression and antigen-specific T-cell responses. Cancer Res.

[37] Rodriguez PC, Zea AH, DeSalvo J, Culotta KS, Zabaleta J, Quiceno DG (2003). L-arginine consumption by macrophages modulates the expression of CD3 zeta chain in T lymphocytes. J Immunol.

[38] Li J, Wang L, Chen X, Li L, Li Y, Ping Y (2017). CD39/CD73 upregulation on myeloid-derived suppressor cells via TGF-beta-mTOR-HIF-1 signaling in patients with non-small cell lung cancer. Oncoimmunology.

[39] Yamauchi Y, Safi S, Blattner C, Rathinasamy A, Umansky L, Juenger S (2018). Circulating and Tumor Myeloid-derived Suppressor Cells in Resectable Non-Small Cell Lung Cancer. Am J Respir Crit Care Med.

[40] Guerriero JL (2018). Macrophages: The Road Less Traveled, Changing Anticancer Therapy. Trends Mol Med.

[41] Mantovani A, Allavena P, Sica A, Balkwill F (2008). Cancer-related inflammation. Nature.

[42] Franklin RA, Liao W, Sarkar A, Kim MV, Bivona MR, Liu K (2014). The cellular and molecular origin of tumor-associated macrophages. Science.

[43] Wynn TA (2015). Type 2 cytokines: mechanisms and therapeutic strategies. Nat Rev Immunol.

[44] Gordon S (2003). Alternative activation of macrophages. Nat Rev Immunol.

[45] Liu XL, Pan Q, Cao HX, Xin FZ, Zhao ZH, Yang RX (2020). Lipotoxic Hepatocyte-Derived Exosomal MicroRNA 192-5p Activates Macrophages Through Rictor/Akt/Forkhead Box Transcription Factor O1 Signaling in Nonalcoholic Fatty Liver Disease. Hepatology.

[46] Ye H, Zhou Q, Zheng S, Li G, Lin Q, Wei L (2018). Tumor-associated macrophages promote progression and the Warburg effect via CCL18/NF-kB/VCAM-1 pathway in pancreatic ductal adenocarcinoma. Cell Death Dis.

[47] Wang Q, Lu Y, Li R, Jiang Y, Zheng Y, Qian J (2018). Therapeutic effects of CSF1R-blocking antibodies in multiple myeloma. Leukemia.

[48] Georgoudaki AM, Prokopec KE, Boura VF, Hellqvist E, Sohn S, Ostling J (2016). Reprogramming Tumor-Associated Macrophages by Antibody Targeting Inhibits Cancer Progression and Metastasis. Cell Rep.

[49] Mantovani A, Locati M (2016). Macrophage Metabolism Shapes Angiogenesis in Tumors. Cell Metab.

[50] Yan H, Dong M, Liu X, Shen Q, He D, Huang X (2019). Multiple myeloma cell-derived IL-32gamma increases the immunosuppressive function of macrophages by promoting indoleamine 2,3-dioxygenase (IDO) expression. Cancer Lett.

[51] He L, Jhong JH, Chen Q, Huang KY, Strittmatter K, Kreuzer J (2021). Global characterization of macrophage polarization mechanisms and identification of M2-type polarization inhibitors. Cell Rep.

[52] Yang J, Zhao Y, Zhang L, Fan H, Qi C, Zhang K (2018). RIPK3/MLKL-Mediated Neuronal Necroptosis Modulates the M1/M2 Polarization of Microglia/Macrophages in the Ischemic Cortex. Cereb Cortex.

[53] Yin C, Han Q, Xu D, Zheng B, Zhao X, Zhang J (2019). SALL4-mediated upregulation of exosomal miR-146a-5p drives T-cell exhaustion by M2 tumor-associated macrophages in HCC. Oncoimmunology.

[54] Kuang DM, Peng C, Zhao Q, Wu Y, Chen MS, Zheng L (2010). Activated monocytes in peritumoral stroma of hepatocellular carcinoma promote expansion of memory T helper 17 cells. Hepatology.

[55] Wang D, Yang L, Yue D, Cao L, Li L, Wang D (2019). Macrophage-derived CCL22 promotes an immunosuppressive tumor microenvironment via IL-8 in malignant pleural effusion. Cancer Lett.

[56] Mamrot J, Balachandran S, Steele EJ, Lindley RA (2019). Molecular model linking Th2 polarized M2 tumour-associated macrophages with deaminase-mediated cancer progression mutation signatures. Scand J Immunol.

[57] Movahedi K, Laoui D, Gysemans C, Baeten M, Stange G, Van den Bossche J (2010). Different tumor microenvironments contain functionally distinct subsets of macrophages derived from Ly6C(high) monocytes. Cancer Res.

[58] Yan W, Liu X, Ma H, Zhang H, Song X, Gao L (2015). Tim-3 fosters HCC development by enhancing TGF-beta-mediated alternative activation of macrophages. Gut.

[59] Wan S, Zhao E, Kryczek I, Vatan L, Sadovskaya A, Ludema G (2014). Tumor-associated macrophages produce interleukin 6 and signal via STAT3 to promote expansion of human hepatocellular carcinoma stem cells. Gastroenterology.

[60] Dong P, Ma L, Liu L, Zhao G, Zhang S, Dong L (2016). CD86(+)/CD206(+), Diametrically Polarized Tumor-Associated Macrophages, Predict Hepatocellular Carcinoma Patient Prognosis. Int J Mol Sci.

[61] Mehta AK, Kadel S, Townsend MG, Oliwa M, Guerriero JL (2021). Macrophage Biology and Mechanisms of Immune Suppression in Breast Cancer. Front Immunol.

[62] Ohue Y, Nishikawa H (2019). Regulatory T (Treg) cells in cancer: Can Treg cells be a new therapeutic target?. Cancer Sci.

[63] Chaudhary B, Elkord E (2016). Regulatory T Cells in the Tumor Microenvironment and Cancer Progression: Role and Therapeutic Targeting. Vaccines (Basel).

[64] Togashi Y, Shitara K, Nishikawa H (2019). Regulatory T cells in cancer immunosuppression - implications for anticancer therapy. Nat Rev Clin Oncol.

[65] Cao X, Cai SF, Fehniger TA, Song J, Collins LI, Piwnica-Worms DR (2007). Granzyme B and perforin are important for regulatory T cell-mediated suppression of tumor clearance. Immunity.

[66] Sarvaria A, Madrigal JA, Saudemont A (2017). B cell regulation in cancer and anti-tumor immunity. Cell Mol Immunol.

[67] Rosser EC, Mauri C (2015). Regulatory B cells: origin, phenotype, and function. Immunity.

[68] Zhou X, Su YX, Lao XM, Liang YJ, Liao GQ (2016). CD19(+)IL-10(+) regulatory B cells affect survival of tongue squamous cell carcinoma patients and induce resting CD4(+) T cells to CD4(+)Foxp3(+) regulatory T cells. Oral Oncol.

[69] Wei X, Jin Y, Tian Y, Zhang H, Wu J, Lu W (2016). Regulatory B cells contribute to the impaired antitumor immunity in ovarian cancer patients. Tumour Biol.

[70] Zhang L, Tai YT, Ho M, Xing L, Chauhan D, Gang A (2017). Regulatory B cell-myeloma cell interaction confers immunosuppression and promotes their survival in the bone marrow milieu. Blood Cancer J.

[71] Michaeli J, Shaul ME, Mishalian I, Hovav AH, Levy L, Zolotriov L (2017). Tumor-associated neutrophils induce apoptosis of non-activated CD8 T-cells in a TNFalpha and NO-dependent mechanism, promoting a tumor-supportive environment. Oncoimmunology.

[72] Powell D, Lou M, Barros BF, Huttenlocher A (2018). Cxcr1 mediates recruitment of neutrophils and supports proliferation of tumor-initiating astrocytes in vivo. Sci Rep.

[73] Andzinski L, Kasnitz N, Stahnke S, Wu CF, Gereke M, von Kockritz-Blickwede M (2016). Type I IFNs induce anti-tumor polarization of tumor associated neutrophils in mice and human. Int J Cancer.

[74] Nicolas-Avila JA, Adrover JM, Hidalgo A (2017). Neutrophils in Homeostasis, Immunity, and Cancer. Immunity.

[75] Jeyakumar G, Kim S, Bumma N, Landry C, Silski C, Suisham S (2017). Neutrophil lymphocyte ratio and duration of prior anti-angiogenic therapy as biomarkers in metastatic RCC receiving immune checkpoint inhibitor therapy. J Immunother Cancer.

[76] Ameratunga M, Chenard-Poirier M, Moreno CI, Pedregal M, Lui A, Dolling D (2018). Neutrophil-lymphocyte ratio kinetics in patients with advanced solid tumours on phase I trials of PD-1/PD-L1 inhibitors. Eur J Cancer.

[77] Komi DEA, Redegeld FA (2020). Role of Mast Cells in Shaping the Tumor Microenvironment. Clin Rev Allergy Immunol.

[78] Majorini MT, Cancila V, Rigoni A, Botti L, Dugo M, Triulzi T (2020). Infiltrating Mast Cell-Mediated Stimulation of Estrogen Receptor Activity in Breast Cancer Cells Promotes the Luminal Phenotype. Cancer Res.

[79] Varricchi G, Galdiero MR, Loffredo S, Marone G, Iannone R, Marone G (2017). Are Mast Cells MASTers in Cancer?. Front Immunol.

[80] Leveque E, Rouch A, Syrykh C, Mazieres J, Brouchet L, Valitutti S (2022). Phenotypic and Histological Distribution Analysis Identify Mast Cell Heterogeneity in Non-Small Cell Lung Cancer. Cancers (Basel).

[81] Oldford SA, Marshall JS (2015). Mast cells as targets for immunotherapy of solid tumors. Mol Immunol.

[82] Makino K, Long MD, Kajihara R, Matsueda S, Oba T, Kanehira K (2022). Generation of cDC-like cells from human induced pluripotent stem cells via Notch signaling. J Immunother Cancer.

[83] Scarlett UK, Rutkowski MR, Rauwerdink AM, Fields J, Escovar-Fadul X, Baird J (2012). Ovarian cancer progression is controlled by phenotypic changes in dendritic cells. J Exp Med.

[84] Laoui D, Keirsse J, Morias Y, Van Overmeire E, Geeraerts X, Elkrim Y (2016). The tumour microenvironment harbours ontogenically distinct dendritic cell populations with opposing effects on tumour immunity. Nat Commun.

[85] Wculek SK, Cueto FJ, Mujal AM, Melero I, Krummel MF, Sancho D (2020). Dendritic cells in cancer immunology and immunotherapy. Nat Rev Immunol.

[86] Hegde S, Krisnawan VE, Herzog BH, Zuo C, Breden MA, Knolhoff BL (2020). Dendritic Cell Paucity Leads to Dysfunctional Immune Surveillance in Pancreatic Cancer. Cancer Cell.

[87] Lin JH, Huffman AP, Wattenberg MM, Walter DM, Carpenter EL, Feldser DM (2020). Type 1 conventional dendritic cells are systemically dysregulated early in pancreatic carcinogenesis. J Exp Med.

[88] Ruiz DGM, Bresnahan E, Molina-Sanchez P, Lindblad KE, Maier B, Sia D (2019). beta-Catenin Activation Promotes Immune Escape and Resistance to Anti-PD-1 Therapy in Hepatocellular Carcinoma. Cancer Discov.

[89] Lin A, Schildknecht A, Nguyen LT, Ohashi PS (2010). Dendritic cells integrate signals from the tumor microenvironment to modulate immunity and tumor growth. Immunol Lett.

[90] de Mingo PA, Gardner A, Hiebler S, Soliman H, Rugo HS, Krummel MF (2018). TIM-3 Regulates CD103(+) Dendritic Cell Function and Response to Chemotherapy in Breast Cancer. Cancer Cell.

[91] Monti M, Vescovi R, Consoli F, Farina D, Moratto D, Berruti A (2020). Plasmacytoid Dendritic Cell Impairment in Metastatic Melanoma by Lactic Acidosis. Cancers (Basel).

[92] Gabrilovich D (2004). Mechanisms and functional significance of tumour-induced dendritic-cell defects. Nat Rev Immunol.

[93] Leonard WJ, Lin JX, O'Shea JJ (2019). The gammac Family of Cytokines: Basic Biology to Therapeutic Ramifications. Immunity.

[94] Zhou J, Jin L, Wang F, Zhang Y, Liu B, Zhao T (2019). Chimeric antigen receptor T (CAR-T) cells expanded with IL-7/IL-15 mediate superior antitumor effects. Protein Cell.

[95] Kaplanski G (2018). Interleukin-18: Biological properties and role in disease pathogenesis. Immunol Rev.

[96] Park S, Cheon S, Cho D (2007). The dual effects of interleukin-18 in tumor progression. Cell Mol Immunol.

[97] Waldmann TA (2018). Cytokines in Cancer Immunotherapy. Cold Spring Harb Perspect Biol.

[98] Anderson AC, Joller N, Kuchroo VK (2016). Lag-3, Tim-3, and TIGIT: Co-inhibitory Receptors with Specialized Functions in Immune Regulation. Immunity.

[99] Han Y, Chen Z, Yang Y, Jiang Z, Gu Y, Liu Y (2014). Human CD14+ CTLA-4+ regulatory dendritic cells suppress T-cell response by cytotoxic T-lymphocyte antigen-4-dependent IL-10 and indoleamine-2,3-dioxygenase production in hepatocellular carcinoma. Hepatology.

[100] Peng D, Kryczek I, Nagarsheth N, Zhao L, Wei S, Wang W (2015). Epigenetic silencing of TH1-type chemokines shapes tumour immunity and immunotherapy. Nature.

[101] Tokunaga R, Zhang W, Naseem M, Puccini A, Berger MD, Soni S (2018). CXCL9, CXCL10, CXCL11/CXCR3 axis for immune activation - A target for novel cancer therapy. Cancer Treat Rev.

[102] Kryczek I, Lange A, Mottram P, Alvarez X, Cheng P, Hogan M (2005). CXCL12 and vascular endothelial growth factor synergistically induce neoangiogenesis in human ovarian cancers. Cancer Res.

[103] Ciummo SL, D'Antonio L, Sorrentino C, Fieni C, Lanuti P, Stassi G (2021). The C-X-C Motif Chemokine Ligand 1 Sustains Breast Cancer Stem Cell Self-Renewal and Promotes Tumor Progression and Immune Escape Programs. Front Cell Dev Biol.

[104] Cheng JT, Deng YN, Yi HM, Wang GY, Fu BS, Chen WJ (2016). Hepatic carcinoma-associated fibroblasts induce IDO-producing regulatory dendritic cells through IL-6-mediated STAT3 activation. Oncogenesis.

[105] Chiu DK, Xu IM, Lai RK, Tse AP, Wei LL, Koh HY (2016). Hypoxia induces myeloid-derived suppressor cell recruitment to hepatocellular carcinoma through chemokine (C-C motif) ligand 26. Hepatology.

[106] Allard B, Allard D, Buisseret L, Stagg J (2020). The adenosine pathway in immuno-oncology. Nat Rev Clin Oncol.

[107] Fong L, Hotson A, Powderly JD, Sznol M, Heist RS, Choueiri TK (2020). Adenosine 2A Receptor Blockade as an Immunotherapy for Treatment-Refractory Renal Cell Cancer. Cancer Discov.

[108] Masoumi E, Jafarzadeh L, Mirzaei HR, Alishah K, Fallah-Mehrjardi K, Rostamian H (2020). Genetic and pharmacological targeting of A2a receptor improves function of anti-mesothelin CAR T cells. J Exp Clin Cancer Res.

[109] Giuffrida L, Sek K, Henderson MA, Lai J, Chen AXY, Meyran D (2021). CRISPR/Cas9 mediated deletion of the adenosine A2A receptor enhances CAR T cell efficacy. Nat Commun.

[110] Newick K, O'Brien S, Moon E, Albelda SM (2017). CAR T Cell Therapy for Solid Tumors. Annu Rev Med.

[111] Choe JH, Watchmaker PB, Simic MS, Gilbert RD, Li AW, Krasnow NA (2021). SynNotch-CAR T cells overcome challenges of specificity, heterogeneity, and persistence in treating glioblastoma. Sci Transl Med.

[112] Majzner RG, Mackall CL (2018). Tumor Antigen Escape from CAR T-cell Therapy. Cancer Discov.

[113] Majzner RG, Heitzeneder S, Mackall CL (2017). Harnessing the Immunotherapy Revolution for the Treatment of Childhood Cancers. Cancer Cell.

[114] Sotillo E, Barrett DM, Black KL, Bagashev A, Oldridge D, Wu G (2015). Convergence of Acquired Mutations and Alternative Splicing of CD19 Enables Resistance to CART-19 Immunotherapy. Cancer Discov.

[115] Tirtakusuma R, Szoltysek K, Milne P, Grinev V, Ptasinska A, Chin PS (2022). Epigenetic regulator genes direct lineage switching in MLL/AF4 leukaemia. Blood.

[116] Fry TJ, Shah NN, Orentas RJ, Stetler-Stevenson M, Yuan CM, Ramakrishna S (2018). CD22-targeted CAR T cells induce remission in B-ALL that is naive or resistant to CD19-targeted CAR immunotherapy. Nat Med.

[117] Tumino N, Weber G, Besi F, Del BF, Bertaina V, Paci P (2021). Polymorphonuclear myeloid-derived suppressor cells impair the anti-tumor efficacy of GD2.CAR T-cells in patients with neuroblastoma. J Hematol Oncol.

[118] Nalawade SA, Shafer P, Bajgain P, McKenna MK, Ali A, Kelly L (2021). Selectively targeting myeloid-derived suppressor cells through TRAIL receptor 2 to enhance the efficacy of CAR T cell therapy for treatment of breast cancer. J Immunother Cancer.

[119] Wu X, Luo H, Shi B, Di S, Sun R, Su J (2019). Combined Antitumor Effects of Sorafenib and GPC3-CAR T Cells in Mouse Models of Hepatocellular Carcinoma. Mol Ther.

[120] Wang X, Walter M, Urak R, Weng L, Huynh C, Lim L (2018). Lenalidomide Enhances the Function of CS1 Chimeric Antigen Receptor-Redirected T Cells Against Multiple Myeloma. Clin Cancer Res.

[121] Sun R, Luo H, Su J, Di S, Zhou M, Shi B (2021). Olaparib Suppresses MDSC Recruitment via SDF1alpha/CXCR4 Axis to Improve the Anti-tumor Efficacy of CAR-T Cells on Breast Cancer in Mice. Mol Ther.

[122] Luo W, Napoleon JV, Zhang F, Lee YG, Wang B, Putt KS (2022). Repolarization of Tumor-Infiltrating Myeloid Cells for Augmentation of CAR T Cell Therapies. Front Immunol.

[123] Roy AG, Robinson JM, Sharma P, Rodriguez-Garcia A, Poussin MA, Nickerson-Nutter C (2021). Folate Receptor Beta as a Direct and Indirect Target for Antibody-Based Cancer Immunotherapy. Int J Mol Sci.

[124] Rodriguez-Garcia A, Lynn RC, Poussin M, Eiva MA, Shaw LC, O'Connor RS (2021). CAR-T cell-mediated depletion of immunosuppressive tumor-associated macrophages promotes endogenous antitumor immunity and augments adoptive immunotherapy. Nat Commun.

[125] Zhang P, Zhao S, Wu C, Li J, Li Z, Wen C (2018). Effects of CSF1R-targeted chimeric antigen receptor-modified NK92MI & T cells on tumor-associated macrophages. Immunotherapy.

[126] Arce VF, Furness AJS, Solomon I, Joshi K, Mekkaoui L, Lesko MH (2017). Fc-Optimized Anti-CD25 Depletes Tumor-Infiltrating Regulatory T Cells and Synergizes with PD-1 Blockade to Eradicate Established Tumors. Immunity.

[127] Suryadevara CM, Desai R, Farber SH, Choi BD, Swartz AM, Shen SH (2019). Preventing Lck Activation in CAR T Cells Confers Treg Resistance but Requires 4-1BB Signaling for Them to Persist and Treat Solid Tumors in Nonlymphodepleted Hosts. Clin Cancer Res.

[128] Fraietta JA, Lacey SF, Orlando EJ, Pruteanu-Malinici I, Gohil M, Lundh S (2018). Determinants of response and resistance to CD19 chimeric antigen receptor (CAR) T cell therapy of chronic lymphocytic leukemia. Nat Med.

[129] Yang CL, Jiang NG, Zhang L, Shen K, Wu Y (2021). Relapsed/refractory multiple myeloma-transformed plasma-cell leukemia successfully treated with daratumumab followed by autologous stem cell transplantation. Ther Adv Hematol.

[130] Jin C, Ma J, Ramachandran M, Yu D, Essand M (2022). CAR T cells expressing a bacterial virulence factor trigger potent bystander antitumour responses in solid cancers. Nat Biomed Eng.

[131] Chmielewski M, Kopecky C, Hombach AA, Abken H (2011). IL-12 release by engineered T cells expressing chimeric antigen receptors can effectively Muster an antigen-independent macrophage response on tumor cells that have shut down tumor antigen expression. Cancer Res.

[132] Chi X, Yang P, Zhang E, Gu J, Xu H, Li M (2019). Significantly increased anti-tumor activity of carcinoembryonic antigen-specific chimeric antigen receptor T cells in combination with recombinant human IL-12. Cancer Med.

[133] Hu B, Ren J, Luo Y, Keith B, Young RM, Scholler J (2017). Augmentation of Antitumor Immunity by Human and Mouse CAR T Cells Secreting IL-18. Cell Rep.

[134] Kunert A, Chmielewski M, Wijers R, Berrevoets C, Abken H, Debets R (2017). Intra-tumoral production of IL18, but not IL12, by TCR-engineered T cells is non-toxic and counteracts immune evasion of solid tumors. Oncoimmunology.

[135] Ma X, Shou P, Smith C, Chen Y, Du H, Sun C (2020). Interleukin-23 engineering improves CAR T cell function in solid tumors. Nat Biotechnol.

[136] Lanitis E, Rota G, Kosti P, Ronet C, Spill A, Seijo B (2021). Optimized gene engineering of murine CAR-T cells reveals the beneficial effects of IL-15 coexpression. J Exp Med.

[137] Adachi K, Kano Y, Nagai T, Okuyama N, Sakoda Y, Tamada K (2018). IL-7 and CCL19 expression in CAR-T cells improves immune cell infiltration and CAR-T cell survival in the tumor. Nat Biotechnol.

[138] Wang Y, Jiang H, Luo H, Sun Y, Shi B, Sun R (2019). An IL-4/21 Inverted Cytokine Receptor Improving CAR-T Cell Potency in Immunosuppressive Solid-Tumor Microenvironment. Front Immunol.

[139] Chang ZL, Lorenzini MH, Chen X, Tran U, Bangayan NJ, Chen YY (2018). Rewiring T-cell responses to soluble factors with chimeric antigen receptors. Nat Chem Biol.

[140] Kloss CC, Lee J, Zhang A, Chen F, Melenhorst JJ, Lacey SF (2018). Dominant-Negative TGF-beta Receptor Enhances PSMA-Targeted Human CAR T Cell Proliferation And Augments Prostate Cancer Eradication. Mol Ther.

[141] Hou AJ, Chang ZL, Lorenzini MH, Zah E, Chen YY (2018). TGF-beta-responsive CAR-T cells promote anti-tumor immune function. Bioeng Transl Med.

[142] Mariathasan S, Turley SJ, Nickles D, Castiglioni A, Yuen K, Wang Y (2018). TGFbeta attenuates tumour response to PD-L1 blockade by contributing to exclusion of T cells. Nature.

[143] Mohammed S, Sukumaran S, Bajgain P, Watanabe N, Heslop HE, Rooney CM (2017). Improving Chimeric Antigen Receptor-Modified T Cell Function by Reversing the Immunosuppressive Tumor Microenvironment of Pancreatic Cancer. Mol Ther.

[144] Leen AM, Sukumaran S, Watanabe N, Mohammed S, Keirnan J, Yanagisawa R (2014). Reversal of tumor immune inhibition using a chimeric cytokine receptor. Mol Ther.

[145] Roth TL, Li PJ, Blaeschke F, Nies JF, Apathy R, Mowery C (2020). Pooled Knockin Targeting for Genome Engineering of Cellular Immunotherapies. Cell.

[146] Wenthe J, Naseri S, Labani-Motlagh A, Enblad G, Wikstrom KI, Eriksson E (2021). Boosting CAR T-cell responses in lymphoma by simultaneous targeting of CD40/4-1BB using oncolytic viral gene therapy. Cancer Immunol Immunother.

[147] Jin L, Tao H, Karachi A, Long Y, Hou AY, Na M (2019). CXCR1- or CXCR2-modified CAR T cells co-opt IL-8 for maximal antitumor efficacy in solid tumors. Nat Commun.

[148] Liu G, Rui W, Zheng H, Huang D, Yu F, Zhang Y (2020). CXCR2-modified CAR-T cells have enhanced trafficking ability that improves treatment of hepatocellular carcinoma. Eur J Immunol.

[149] Whilding LM, Halim L, Draper B, Parente-Pereira AC, Zabinski T, Davies DM (2019). CAR T-Cells Targeting the Integrin alphavbeta6 and Co-Expressing the Chemokine Receptor CXCR2 Demonstrate Enhanced Homing and Efficacy against Several Solid Malignancies. Cancers (Basel).

[150] Perera LP, Zhang M, Nakagawa M, Petrus MN, Maeda M, Kadin ME (2017). Chimeric antigen receptor modified T cells that target chemokine receptor CCR4 as a therapeutic modality for T-cell malignancies. Am J Hematol.

[151] Wang Y, Wang J, Yang X, Yang J, Lu P, Zhao L (2021). Chemokine Receptor CCR2b Enhanced Anti-tumor Function of Chimeric Antigen Receptor T Cells Targeting Mesothelin in a Non-small-cell Lung Carcinoma Model. Front Immunol.

[152] Li G, Zhang Q, Han Z, Zhu Y, Shen H, Liu Z (2021). IL-7 and CCR2b Co-Expression-Mediated Enhanced CAR-T Survival and Infiltration in Solid Tumors. Front Oncol.

[153] Newick K, O'Brien S, Sun J, Kapoor V, Maceyko S, Lo A (2016). Augmentation of CAR T-cell Trafficking and Antitumor Efficacy by Blocking Protein Kinase A Localization. Cancer Immunol Res.

[154] Lanitis E, Kosti P, Ronet C, Cribioli E, Rota G, Spill A (2021). VEGFR-2 redirected CAR-T cells are functionally impaired by soluble VEGF-A competition for receptor binding. J Immunother Cancer.

[155] Fitzgerald JC, Weiss SL, Maude SL, Barrett DM, Lacey SF, Melenhorst JJ (2017). Cytokine Release Syndrome After Chimeric Antigen Receptor T Cell Therapy for Acute Lymphoblastic Leukemia. Crit Care Med.

[156] Lee DW, Santomasso BD, Locke FL, Ghobadi A, Turtle CJ, Brudno JN (2019). ASTCT Consensus Grading for Cytokine Release Syndrome and Neurologic Toxicity Associated with Immune Effector Cells. Biol Blood Marrow Transplant.

[157] Brudno JN, Kochenderfer JN (2016). Toxicities of chimeric antigen receptor T cells: recognition and management. Blood.

[158] Kennedy LB, Salama AKS (2020). A review of cancer immunotherapy toxicity. CA Cancer J Clin.

[159] Grosser R, Cherkassky L, Chintala N, Adusumilli PS (2019). Combination Immunotherapy with CAR T Cells and Checkpoint Blockade for the Treatment of Solid Tumors. Cancer Cell.

[160] Gargett T, Yu W, Dotti G, Yvon ES, Christo SN, Hayball JD (2016). GD2-specific CAR T Cells Undergo Potent Activation and Deletion Following Antigen Encounter but can be Protected From Activation-induced Cell Death by PD-1 Blockade. Mol Ther.

[161] Cherkassky L, Morello A, Villena-Vargas J, Feng Y, Dimitrov DS, Jones DR (2016). Human CAR T cells with cell-intrinsic PD-1 checkpoint blockade resist tumor-mediated inhibition. J Clin Invest.

[162] Li S, Siriwon N, Zhang X, Yang S, Jin T, He F (2017). Enhanced Cancer Immunotherapy by Chimeric Antigen Receptor-Modified T Cells Engineered to Secrete Checkpoint Inhibitors. Clin Cancer Res.

[163] Heczey A, Louis CU, Savoldo B, Dakhova O, Durett A, Grilley B (2017). CAR T Cells Administered in Combination with Lymphodepletion and PD-1 Inhibition to Patients with Neuroblastoma. Mol Ther.

[164] Chong EA, Melenhorst JJ, Lacey SF, Ambrose DE, Gonzalez V, Levine BL (2017). PD-1 blockade modulates chimeric antigen receptor (CAR)-modified T cells: refueling the CAR. Blood.

[165] Menger L, Sledzinska A, Bergerhoff K, Vargas FA, Smith J, Poirot L (2016). TALEN-Mediated Inactivation of PD-1 in Tumor-Reactive Lymphocytes Promotes Intratumoral T-cell Persistence and Rejection of Established Tumors. Cancer Res.

[166] Hu W, Zi Z, Jin Y, Li G, Shao K, Cai Q (2019). CRISPR/Cas9-mediated PD-1 disruption enhances human mesothelin-targeted CAR T cell effector functions. Cancer Immunol Immunother.

[167] Ren J, Liu X, Fang C, Jiang S, June CH, Zhao Y (2017). Multiplex Genome Editing to Generate Universal CAR T Cells Resistant to PD1 Inhibition. Clin Cancer Res.

[168] Jung IY, Kim YY, Yu HS, Lee M, Kim S, Lee J (2018). CRISPR/Cas9-Mediated Knockout of DGK Improves Antitumor Activities of Human T Cells. Cancer Res.

[169] Sakuishi K, Apetoh L, Sullivan JM, Blazar BR, Kuchroo VK, Anderson AC (2010). Targeting Tim-3 and PD-1 pathways to reverse T cell exhaustion and restore anti-tumor immunity. J Exp Med.

[170] Shapiro M, Herishanu Y, Katz BZ, Dezorella N, Sun C, Kay S (2017). Lymphocyte activation gene 3: a novel therapeutic target in chronic lymphocytic leukemia. Haematologica.

[171] Klampatsa A, Leibowitz MS, Sun J, Liousia M, Arguiri E, Albelda SM (2020). Analysis and Augmentation of the Immunologic Bystander Effects of CAR T Cell Therapy in a Syngeneic Mouse Cancer Model. Mol Ther Oncolytics.

[172] Rafiq S, Yeku OO, Jackson HJ, Purdon TJ, van Leeuwen DG, Drakes DJ (2018). Targeted delivery of a PD-1-blocking scFv by CAR-T cells enhances anti-tumor efficacy in vivo. Nat Biotechnol.

[173] Hegde M, Mukherjee M, Grada Z, Pignata A, Landi D, Navai SA (2016). Tandem CAR T cells targeting HER2 and IL13Ralpha2 mitigate tumor antigen escape. J Clin Invest.

[174] Li KX, Wu HY, Pan WY, Guo MQ, Qiu DZ, He YJ (2022). A novel approach for relapsed/refractory FLT3(mut+) acute myeloid leukaemia: synergistic effect of the combination of bispecific FLT3scFv/NKG2D-CAR T cells and gilteritinib. Mol Cancer.

[175] Wang H, Wang X, Ye X, Ju Y, Cao N, Wang S (2022). Nonviral mcDNA-mediated bispecific CAR T cells kill tumor cells in an experimental mouse model of hepatocellular carcinoma. BMC Cancer.

[176] Yin Y, Rodriguez JL, Li N, Thokala R, Nasrallah MP, Hu L (2022). Locally secreted BiTEs complement CAR T cells by enhancing killing of antigen heterogeneous solid tumors. Mol Ther.

[177] Choi BD, Yu X, Castano AP, Bouffard AA, Schmidts A, Larson RC (2019). CAR-T cells secreting BiTEs circumvent antigen escape without detectable toxicity. Nat Biotechnol.

[178] Vaidya A, Doherty E, Wu X, Huang S, Hebbar N, Thanekar U (2022). Improving the anti-acute myeloid leukemia activity of CD123-specific Engager T cells by MyD88 and CD40 costimulation. Haematologica.

[179] Binnewies M, Roberts EW, Kersten K, Chan V, Fearon DF, Merad M (2018). Understanding the tumor immune microenvironment (TIME) for effective therapy. Nat Med.

[180] Yang M, Li J, Gu P, Fan X (2021). The application of nanoparticles in cancer immunotherapy: Targeting tumor microenvironment. Bioact Mater.

[181] Luo Y, Chen Z, Sun M, Li B, Pan F, Ma A (2022). IL-12 nanochaperone-engineered CAR T cell for robust tumor-immunotherapy. Biomaterials.

[182] Johnson LR, Lee DY, Eacret JS, Ye D, June CH, Minn AJ (2021). The immunostimulatory RNA RN7SL1 enables CAR-T cells to enhance autonomous and endogenous immune function. Cell.

[183] Hauth F, Ho AY, Ferrone S, Duda DG (2021). Radiotherapy to Enhance Chimeric Antigen Receptor T-Cell Therapeutic Efficacy in Solid Tumors: A Narrative Review. JAMA Oncol.

[184] DeSelm C, Palomba ML, Yahalom J, Hamieh M, Eyquem J, Rajasekhar VK (2018). Low-Dose Radiation Conditioning Enables CAR T Cells to Mitigate Antigen Escape. Mol Ther.

[185] Sim AJ, Jain MD, Figura NB, Chavez JC, Shah BD, Khimani F (2019). Radiation Therapy as a Bridging Strategy for CAR T Cell Therapy With Axicabtagene Ciloleucel in Diffuse Large B-Cell Lymphoma. Int J Radiat Oncol Biol Phys.

[186] Shao F, Long Y, Ji H, Jiang D, Lei P, Lan X (2021). Radionuclide-based molecular imaging allows CAR-T cellular visualization and therapeutic monitoring. Theranostics.

[187] Neelapu SS, Tummala S, Kebriaei P, Wierda W, Gutierrez C, Locke FL (2018). Chimeric antigen receptor T-cell therapy - assessment and management of toxicities. Nat Rev Clin Oncol.

[188] Dieu-Nosjean MC, Giraldo NA, Kaplon H, Germain C, Fridman WH, Sautes-Fridman C (2016). Tertiary lymphoid structures, drivers of the anti-tumor responses in human cancers. Immunol Rev.

[189] Del PN, Shirure VS, Bi Y, Goedegebuure SP, Gholami S, Hughes CCW (2021). Tumor-on-chip modeling of organ-specific cancer and metastasis. Adv Drug Deliv Rev.

[190] Aung A, Kumar V, Theprungsirikul J, Davey SK, Varghese S (2020). An Engineered Tumor-on-a-Chip Device with Breast Cancer-Immune Cell Interactions for Assessing T-cell Recruitment. Cancer Res.

[191] Liu X, Fang J, Huang S, Wu X, Xie X, Wang J (2021). Tumor-on-a-chip: from bioinspired design to biomedical application. Microsyst Nanoeng.

[192] Motazedian A, Bruveris FF, Kumar SV, Schiesser JV, Chen T, Ng ES (2020). Multipotent RAG1+ progenitors emerge directly from haemogenic endothelium in human pluripotent stem cell-derived haematopoietic organoids. Nat Cell Biol.

[193] Sugimura R, Ohta R, Mori C, Li A, Mano T, Sano E (2020). Biomimetic aorta-gonad-Mesonephros-on-a-Chip to study human developmental hematopoiesis. Biomed Microdevices.

[194] Wilson TL, Kim H, Chou CH, Langfitt D, Mettelman RC, Minervina AA (2022). Common trajectories of highly effective CD19-specific CAR T cells identified by endogenous T cell receptor lineages. Cancer Discov.

[195] Klichinsky M, Ruella M, Shestova O, Lu XM, Best A, Zeeman M (2020). Human chimeric antigen receptor macrophages for cancer immunotherapy. Nat Biotechnol.

[196] Li Y, Hermanson DL, Moriarity BS, Kaufman DS (2018). Human iPSC-Derived Natural Killer Cells Engineered with Chimeric Antigen Receptors Enhance Anti-tumor Activity. Cell Stem Cell.

